# Characterization of Aurintricarboxylic Acid (ATA) Interactions with Plasma Transporter Protein and SARS-CoV-2 Viral Targets: Correlation of Functional Activity and Binding Energetics

**DOI:** 10.3390/life12060872

**Published:** 2022-06-10

**Authors:** Conceição A. Minetti, David P. Remeta, Keiji Hashimoto, Radha Bonala, Rajesh Chennamshetti, Xingyu Yin, Miguel Garcia-Diaz, Arthur P. Grollman, Francis Johnson, Viktoriya S. Sidorenko

**Affiliations:** 1 Department of Chemistry and Chemical Biology, Rutgers—The State University of New Jersey, Piscataway, NJ 08854, USA; 2 Department of Pharmacological Sciences, State University of New York at Stony Brook, Stony Brook, NY 11794, USA; keiji.hashimoto@stonybrook.edu (K.H.); radha.bonala@stonybrook.edu (R.B.); rajesh.chennamshetti@stonybrook.edu (R.C.); xingyu.yin@stonybrook.edu (X.Y.); miguel.garcia-diaz@stonybrook.edu (M.G.-D.); arthur.grollman@stonybrook.edu (A.P.G.); 3 Department of Medicine, State University of New York at Stony Brook, Stony Brook, NY 11794, USA; 4 Department of Chemistry, State University of New York at Stony Brook, Stony Brook, NY 11794, USA

**Keywords:** aurintricarboxylic acid (ATA), salicylic acid polymers, methylene-salicylic acid, SARS-CoV-2, RNA-dependent RNA polymerase (RdRp), yeast ribosomes, human serum albumin (HSA), molecular recognition, biological function and viral disease, inhibitor binding, thermodynamics

## Abstract

In an effort to identify functional-energetic correlations leading to the development of efficient anti-SARS-CoV-2 therapeutic agents, we have designed synthetic analogs of aurintricarboxylic acid (ATA), a heterogeneous polymeric mixture of structurally related linear homologs known to exhibit a host of biological properties, including antiviral activity. These derivatives are evaluated for their ability to interact with a plasma transporter protein (human serum albumin), eukaryotic (yeast) ribosomes, and a SARS-CoV-2 target, the RNA-dependent RNA polymerase (RdRp). The resultant data are critical for characterizing drug distribution, bioavailability, and effective inhibition of host and viral targets. Promising lead compounds are selected on the basis of their binding energetics which have been characterized and correlated with functional activities as assessed by inhibition of RNA replication and protein synthesis. Our results reveal that the activity of heterogeneous ATA is mimicked by linear compounds of defined molecular weight, with a dichlorohexamer salicylic-acid derivative exhibiting the highest potency. These findings are instrumental for optimizing the design of structurally defined ATA analogs that fulfill the requirements of an antiviral drug with respect to bioavailability, homogeneity, and potency, thereby expanding the arsenal of therapeutic regimens that are currently available to address the urgent need for effective SARS-CoV-2 treatment strategies.

## 1. Introduction

The emergence of highly infectious life-threatening diseases, as evidenced by the SARS-CoV-2 global pandemic, necessitates effective treatment strategies, including the development and evaluation of novel pharmacological compounds as prospective antiviral therapeutics. Over the past several decades, the Breslauer, Grollman, and Johnson research laboratories have pursued a collaborative synergistic program to elucidate specific structure–function–energetic correlations in systems of biomedical relevance. This comprehensive multiparametric strategy has enabled us to characterize the overall impact of carcinogenic and mutagenic DNA lesions [1,2,3,4,5,6,7,8,9] on nucleic acid recognition [10], replication [11], and repair [10,12] (as reviewed in [13]). Applying the methodology developed and refined during this timeframe towards identifying effective treatment protocols to combat infectious diseases, our laboratories are currently exploring investigational compounds and repurposed molecules to assess their overall therapeutic potential as anti-SARS-CoV-2 inhibitors. The latter includes aurintricarboxylic acid (ATA), a salicylic acid polymer that exhibits a broad range of biological activities, including its efficacy as an antiviral agent [14,15].

The synthesis of ATA traces its origin to 1892 as the primary product of a condensation reaction between salicylic acid and formaldehyde in the presence of sulfuric acid and nitrite ion [16]. Multiple applications have been reported since its discovery, including seminal studies conducted during the 1960s and 1970s [17] that explored ATA as an inhibitor of protein synthesis in eukaryotes while elucidating specific mechanisms of several antiviral and antibiotic compounds at the ribosomal level [18,19,20,21,22,23,24,25,26]. Harnessing insights gained from parallel investigations on emetine [17,18,19,24,27], Grollman and colleagues focused particular attention on the initiation of viral protein synthesis as a target for anti-viral drug design. These researchers demonstrated that triphenylmethane dyes such as ATA inhibit initiation of protein synthesis [21] and prevent the attachment of viral RNA to ribosomes isolated from rabbit reticulocytes [20,23]. Subsequent investigations identified nucleic acid binding proteins as an alternate target for ATA [28] via mechanisms suggested to involve inhibition of nucleic acid interactions with the template binding sites [29,30]. Significantly, the groundbreaking studies conducted by Grollman and associates have inspired the publication of over 750 articles on ATA describing its biomedical applications. A long-standing challenge that remains is to characterize the active principle constituent(s) in the ATA mixture and develop lead compounds for further optimization.

Our approach to antiviral drug design is consistent with these observations as we explore initiation of protein synthesis at the ribosomal level and inhibition of RNA-dependent RNA polymerase (RdRp). Moreover, compounds that are structurally related to ATA and share its mode of action might lead to the design of novel antiviral agents [20,31]. The polymeric heterogeneous nature of ATA mixtures imposes a significant challenge for medicinal chemistry investigations of such compounds. Nevertheless, the ability to inhibit an early event in viral replication suggests that the active principle(s) of this heterogeneous mixture might represent a novel chemical entity. In view of its effective utility as both a protein and nucleic acid synthesis inhibitor, heterogeneous ATA mixtures have been demonstrated to exhibit antiviral activity by inhibiting replication of influenza [26], *coxsackievirus* [32], and SARS-CoV [33]. Recently, screenings of prospective anti-SARS-CoV-2 inhibitors have identified ATA as exhibiting antiviral activity via inhibition of viral cell attachment and invasion by blocking spike protein interactions with ACE2 [34].

Considering the myriad of biological and antiviral properties associated with ATA, identification and resolution of the active component(s) represents a critical hurdle in developing these as lead compounds for further optimization to achieve both a higher potency and selectivity. A feasible and practical alternative is to design synthetic protocols that afford generation of homogeneous lead compounds which mimic and/or exceed the polymeric mixture activity. As part of our ongoing efforts to correlate biological and biophysical properties, we are pursuing leads generated via the design of defined synthetic ATA derivatives that optimize inhibitory activity, antiviral selectivity, bioavailability, and the ability to penetrate cells. The current synthetic scheme to produce ATA involves treatment of salicylic acid with formaldehyde, sulfuric acid, and sodium nitrite resulting in a heterogeneous polymeric mixture [35]. The degree of product heterogeneity is dependent on synthetic conditions such as reactant concentrations, reaction time, and temperature [36,37]. The heterogeneity of ATA preparations has been acknowledged by several investigators [36,38] who have provided detailed information on optimal synthetic routes and product characterization [39,40].

**Scheme 1:**
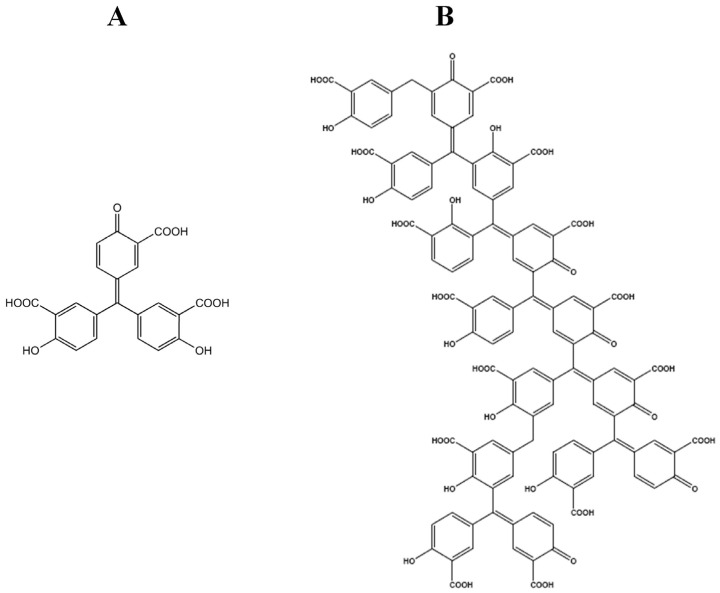
Chemical Structures of Aurintricarboxylic Acid (ATA). (**A**) Monomer (triphenylmethane unit). (**B**) Idealized representative ATA polymer within the heterogeneous mixture (based on [35]).

Although specific efforts have been undertaken to isolate/segregate the active fraction(s) and identify their respective biological properties [41], attempts to purify and characterize a homogeneous ATA species have not succeeded. These observations inevitably lead to the proposition that aurintricarboxylic acid (i.e., monomeric ATA) as depicted in Scheme 1A is in fact an inactive compound [36,41] and its functional activities are conceivably the consequence of “impurities” including formaurindicarboxylic acid and higher molecular weight species [36]. Despite its predominantly polymeric nature, ATA is often regarded as a monomeric triphenylmethane dye that is presumed to represent the active ligand state in molecular docking studies. Consequently, biological properties attributed to ATA are interpreted via computational and prediction methods as arising from specific interactions between the monomer and molecular target(s). The body of experimental evidence accumulated to date supports the notion that ATA biological properties are primarily attributed to compounds of average molecular weight ≥ 2000 [39,42] with a predominant species proposed as illustrated in Scheme 1B.

**Scheme 2:**
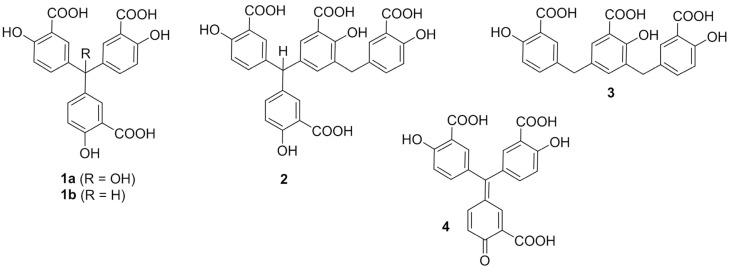
Chemical Structures of ATA Species Isolated from a Fractionated Preparation (based on [35]).

Since the discovery of ATA antiviral properties [20,21], there have been extensive investigations regarding the components responsible for biological activity. Perhaps the most significant studies conducted by Cushman and associates examined both low [39,43] and high [35] molecular weight components of this dyestuff for antiviral activity. These investigators concluded that amongst the former, compounds **2** and **3** (Scheme 2) are the most active against HIV-1 in cultured mammalian cells. Significantly, neither of the methyl esters is active thereby indicating the important role of carboxylic anions. Moreover, the low molecular weight compounds containing a quinomethine residue are inactive. The greatest activity appears in the 7000–12,000 MW range with weight average (M_w_) and number average (M_n_) molecular weights of approximately 2937 and 2547, respectively [35]. Basic matrix–like structures **1a**, **1b** and **4** (Scheme 2) have been proposed [35,39,43] as repeating units in the higher MW polymers yet scant evidence exists to support such chemical structures. Nevertheless, a prominent feature in both series of compounds is the quinomethine group, which accounts for the orange–red color observed in most substances.

Considering the heterogeneity of polymeric ATA mixtures, it is desirable to identify the active component(s) responsible for biological and selective antiviral activities. Our experimental strategy involves the synthesis of defined chemical structures that mimic specific components comprising the heterogeneous mixture. This is accomplished by synthesizing homogeneous linear polymers (i.e., dimers, tetramers, hexamers, octamers, etc.) *in lieu of* triphenylmethane units. It is important to note that when a commercial sample of ATA is reduced by zinc dust in acetic acid to convert the quinomethine groups to salicylic acid residues, antiviral activity is retained in the colorless product. This finding in conjunction with the studies of Cushman and colleagues [35,38,39,40,42,43] infers that biological activity is due primarily to a run of methylene-salicylic residues in the ATA polymers and suggests that the quinomethine groups represented in **4** (Scheme 2) do not contribute to this activity. These observations set the scene for establishing a synthetic strategy in which oligomers comprised solely of methylene-salicylic acid units are evaluated for the purpose of determining whether un-oxidized regions are the primary source of biological activity.

Our systematic multidisciplinary protocol focuses on identifying a prospective lead compound of defined chemical structure and molecular weight that exhibits functional activity comparable to or exceeding the heterogeneous ATA mixture. This study describes the synthesis of a dichlorohexamer that mimics polymeric ATA in terms of its biological activities toward initiation of protein synthesis and SARS-CoV-2 RdRp, yet retains the characteristic features of a lead compound with respect to homogeneity and ADMET qualities. We employ a complementary array of binding assays that utilize a combination of calorimetric and optical techniques to characterize the properties of lead compounds that inhibit RdRp and mammalian protein synthesis in cell-free systems. Parallel experiments profile the binding of heterogeneous polymeric ATA to human serum albumin (HSA), a universal drug-transporter protein in the bloodstream that possesses a unique ability to interact with a myriad of compounds harboring a broad range of hydrophobicities and molecular weights. An accurate assessment of HSA binding energetics is essential, as transporter proteins can diminish the overall bioavailability and/or bioactivity of lead compounds by competing for drug binding to viral or infected host cell receptor sites. Acquisition of the relevant biological and biophysical properties for ATA interactions with antiviral targets facilitates function–energetic correlations. The resultant data serve as a baseline for investigations on small molecule interactions with specific viral targets, including SARS-CoV-2 RdRp and the eukaryotic ribosome.

## 2. Results

### 2.1. Experimental Strategy

This study employs a complementary array of biophysical binding assays that utilize a combination of calorimetric and optical techniques to identify and characterize the properties of aurintricarboxylic acid (ATA) (refer to Scheme 1) as a prospective lead compound. ATA is available commercially or as a component of LOPAC screening libraries (refer to Materials and Methods) and comprises a heterogeneous mixture of oligomeric/polymeric species that exhibit antiviral activity [35,44]. We initially assess the binding properties of ATA to human serum albumin (HSA) as characterization of such interactions provides a measure of plasma bioavailability which can be integrated within ADMET properties to evaluate lead compound pharmacokinetics. Whereas a low binding affinity suggests immediate availability of the administered compound, a high binding affinity infers that HSA may function as a reservoir maintaining homogeneous distribution to the tissues thereby increasing biological lifetimes [45]. Considering its role on overall drug pharmacokinetics [46], HSA may limit and/or control toxicity while modulating metabolic inactivation and elimination through excretory pathways. A secondary yet equally relevant objective underlying initial assessment of HSA binding properties is to identify predominant molecular species in the compound mixture and determine an average molecular weight. The resultant compilation of biophysical properties sets the stage for investigations on ATA derivative interactions with specific host targets including SARS-CoV-2 RdRp which is crucial for viral replication and the 40s ribosomal subunit where initiation of viral protein synthesis occurs.

### 2.2. Biophysical Properties and Binding Energetics

#### 2.2.1. Optical Characterization of ATA–HSA Interactions: Binding Is Accompanied by Fluorescence Quenching and Energy Transfer

**Figure 1:**
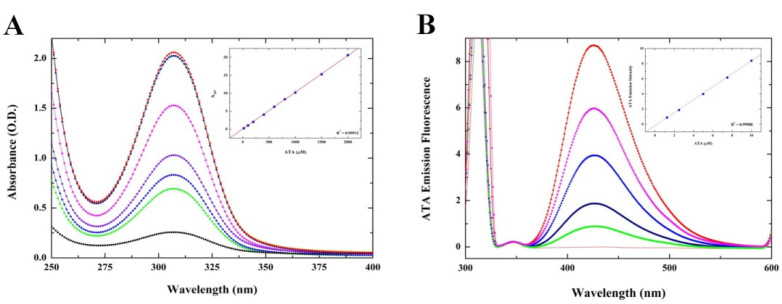
UV/Vis Absorbance and Fluorescence Spectra of the ATA Polymer Mixture. (**A**) Concentration-dependent absorbance spectra and corresponding linear correlation (inset); (**B**) Fluorescence emission profiles exhibiting a maximum intensity at 425 nm that are linearly correlated with increasing ATA concentration (inset).

We have characterized the biophysical and physicochemical properties of ATA by exploring specific optical (i.e., UV/Vis, Fluorescence) profiles. Analysis of the resultant UV/Vis spectrum acquired over the wavelength range of 200–800 nm reveals major and minor absorbance peaks centered at ~310 nm and 528 nm, respectively. The latter is only detected at sufficiently high concentrations (Figure S1) and reflects the presence of higher molecular species which exhibit more extended conjugation relative to the lower molecular weight compounds [35]. Concentration-dependent absorbance (Figure 1A) and fluorescence (Figure 1B) profiles suggest that ATA polymers do not undergo further intermolecular self-association in solution as evidenced by a linear increase of intensity (Figure 1A,B insets). The absorbance peak wavelength (i.e., 310 nm) in the UV/Vis spectrum (Figure 1A) has been selected for excitation of ATA and the resultant emission spectra recorded over 270–600 nm (Figure 1B) with a characteristic maximum emission intensity observed at 425 nm.

**Figure 2:**
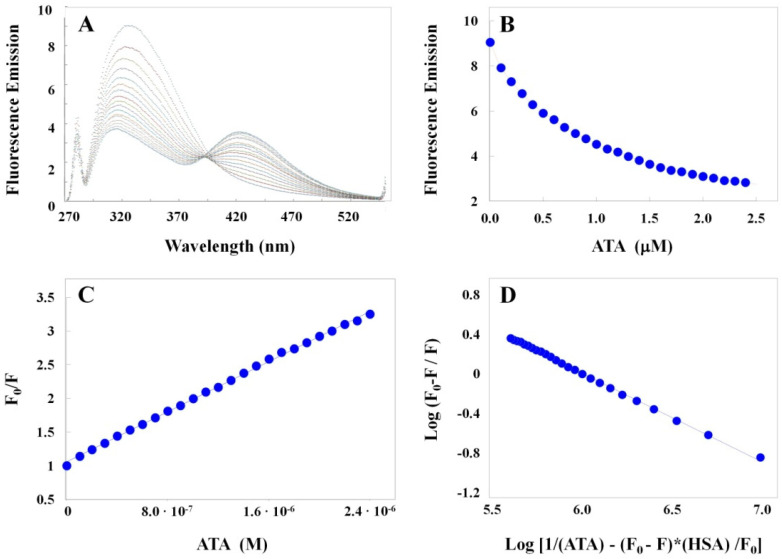
(**A**) Family of HSA Fluorescence Profiles Monitoring ATA-Induced Quenching. The quenching of HSA fluorescence monitored at 325 nm is accompanied by a concomitant increase in ATA emission intensity at 425 nm. Each spectral profile is corrected by subtracting an appropriate reference standard to eliminate the intrinsic fluorescence of ATA upon excitation at 280 nm. (**B**) Emission intensities monitored at 325 nm as a function of ATA concentration. (**C**) Stern–Volmer plot revealing a K_sv_ of 9.2 × 10^5^ M^−1^ (R^2^ = 0.998). (**D**) Double logarithmic plot of the ATA concentration-dependent quenching yielding a single set of binding sites (*n* = 0.9) and an affinity (K_a_) of 5.98 × 10^5^ M^−1^.

A detailed analysis of ATA–HSA interactions monitored via fluorescence spectroscopy reveals that ATA promotes a reduction of the HSA aromatic residue intensity and the resultant quenching is accompanied by fluorescence resonance energy transfer (FRET) that is simultaneously detected at 425 nm. Specifically, when ATA is titrated into HSA and the aromatic residues are excited at 280 nm, one observes a binding-induced quenching of HSA emission intensity at 330 nm and a concomitant increase in ATA fluorescence emission intensity at 425 nm as depicted in Figure 2A. This enhancement is only evident in the presence of HSA, as isolated ATA standards excited at 280 nm do not exhibit an increased intensity at 425 nm (Figure S2). A secondary optical probe of the drug–protein interaction involves monitoring titration of HSA into an ATA solution excited at 310 nm which results in a concentration-dependent enhancement of ATA fluorescence at 425 nm (Figure S3).

The measurable increase in fluorescence intensity exhibited by ATA when excited at 280 nm only occurs in the presence of HSA and presumably arises as a consequence of energy transfer which can be exploited to assess ATA interactions at specific protein binding sites. This observation infers that upon excitation of HSA Tyr and Trp residues, the proximity of ATA to these aromatic residue(s) at the ligand-protein binding site(s) facilitates detection and quantization of the interaction via FRET that can occur from an excited fluorescent donor (HSA) to a suitable acceptor (ATA). Significantly, one observes a characteristic isoemissive wavelength at ~395 nm, which suggests the presence of two distinct emitting species. Exploitation of these unique fluorescent properties represents a useful probe of ligand–receptor interactions and validation of this protocol is a powerful tool for evaluating the interaction of antiviral molecules with SARS-CoV-2 targets.

The HSA fluorescence quenching data monitored at 325 nm as a function of increasing ATA concentrations (Figure 2B) are recast in the form of a Stern–Volmer plot (Figure 2C). A linear Stern–Volmer plot is consistent with static quenching experienced by a single class of fluorophores that is equally accessible to the quencher. The ATA–HSA binding profile has also been analyzed via a double-reciprocal logarithmic plot (Figure 2D). Inspection of the data reveals that this interaction involves a single set of sites (*n* = 0.9) and is characterized by a binding constant (K_b_) of 6.0 × 10^5^ M^−1^ (i.e., K_d_ = 1.7 μM).

#### 2.2.2. Elucidating ATA–HSA Binding Energetics via ITC: Protein-Ligand Complex Formation Is Enthalpy-Driven with Favorable Entropic Contributions

**Figure 3:**
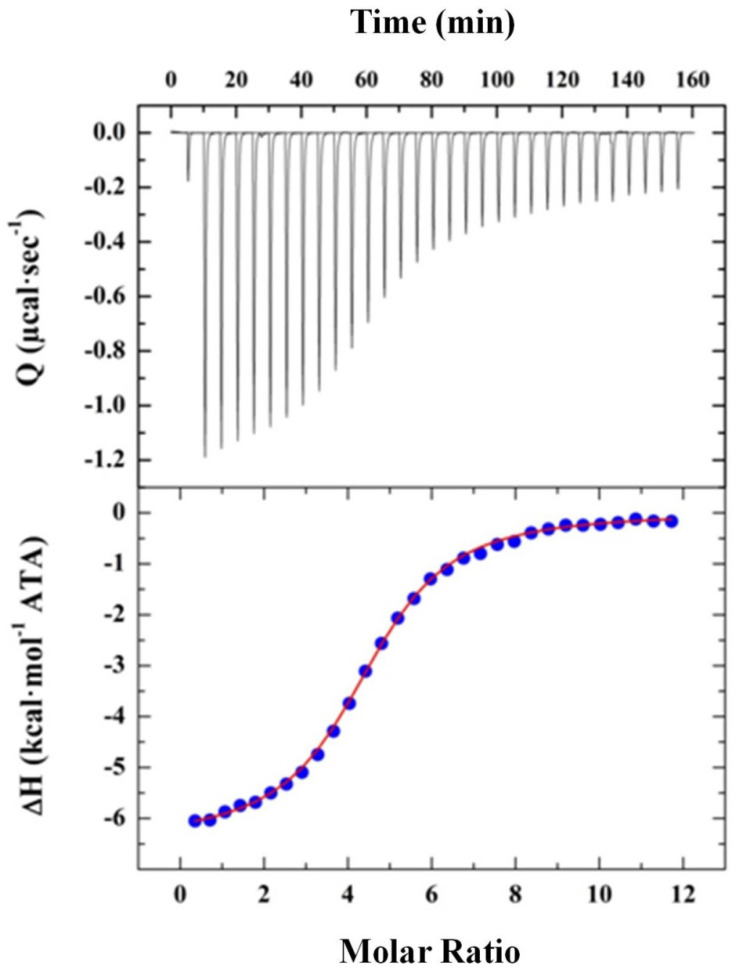
ITC Profile Monitoring ATA–HSA Association. The binding isotherms are acquired at 25.0 °C in 10 mM sodium phosphate buffer (pH 7.4). Titration of HSA (10 μM) with ATA (500 μM) is reflected in the thermogram (top panel), integrated peak areas (blue circles), and corresponding fit (red line) to a single site binding model (bottom panel).

Isothermal Titration Calorimetry (ITC) facilitates characterization of ligand–receptor binding energetics and thereby, furnishes a complete thermodynamic description of the ATA–HSA association process. The standard experimental protocol consists of titrating a concentrated ATA stock solution (500 μM) via thirty successive 10 µL aliquots into an HSA standard (10 μM) employing a 300 s integration period between injections. A representative ITC thermogram reflecting the exothermic reaction accompanying binding of ATA to HSA is depicted in the upper panel of Figure 3. The resultant heats are integrated to yield a binding profile (bottom panel) that is fit via nonlinear least squares analysis to a single site model (red line). Formation of the ATA–HSA complex is characterized by a Gibbs Free Energy (ΔG) of −7.6 kcal·mol^−1^ that is primarily enthalpy driven (ΔH = −6.4 kcal·mol^−1^) with a favorable entropic contribution (TΔS = 1.2 kcal·mol^−1^) and an overall binding affinity (K_a_) of 5.0 × 10^5^ M^−1^. The K_a_ values derived from fluorescence and ITC measurements (i.e., ~5–6 × 10^5^ M^−1^) are in excellent agreement. The enthalpic nature of these interactions is consistent with the polar character of ATA and entropically favorable contributions may be attributed to binding-induced desolvation of the HSA binding site.

Recasting the binding energetics in terms of an ATA monomer (M_w_ = 422.3) as the modular/structural unit (refer to Scheme 1A), the resultant stoichiometric ratio (*n* ~ 4.5) reveals that polymeric ATA preparations comprise an average molecular weight species approximating 2000. These findings suggest that the predominant species are on the order of 4–5 ATA monomeric units. Systematic studies employing well-defined molecular weight compounds (i.e., monomer, dimer, trimer, etc.) should assist in evaluating our working hypothesis regarding the stoichiometric ratio observed. Determining the potential self-assembly of small molecules is pivotal for evaluating their spatial distribution in a cellular environment [47] and a primary factor in assessing the IC_50_ towards a specific target. These non-covalent interactions can be sufficiently ordered to form large assemblies or nanostructures yet may also be responsible for disordered aggregation and precipitation. A control experiment in which the ATA stock solution is diluted into dialysate appears in Figure S4 and reveals negligible reaction heats, thereby confirming that the ATA polymers do not undergo non-covalent intermolecular self-association. Collectively, our results suggest that ATA interacts with one of the HSA subdomains in a single site binding mode with the ligand averaging a molecular weight approximating 2000, which is equivalent to 4–5 ATA monomeric species.

#### 2.2.3. Characterizing HSA Binding of a Dichlorohexamer ATA Analog via ITC: Protein-Ligand Complex Formation Is Enthalpy-Driven with Unfavorable Entropic Contributions

**Scheme 3:**
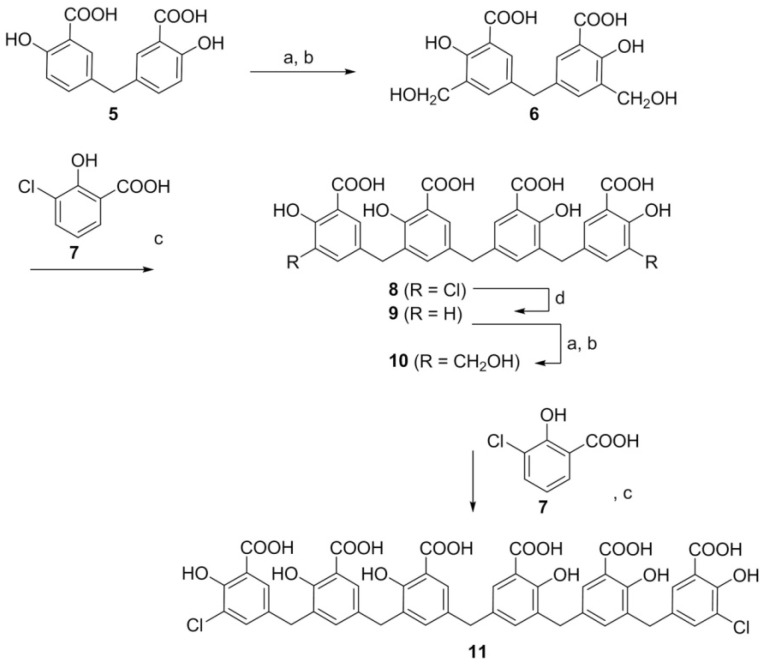
Synthesis of a Chlorinated Polysalicylic Acid Hexamer (Compound **11**) Evaluated as a Prospective Antiviral Therapeutic. Chemical Reagents: (a) hexamethylene tetramine/triethylamine; (b) LiBH_4_; (c) sulfuric acid; (d) 10% PdO, H2.

**Figure 4:**
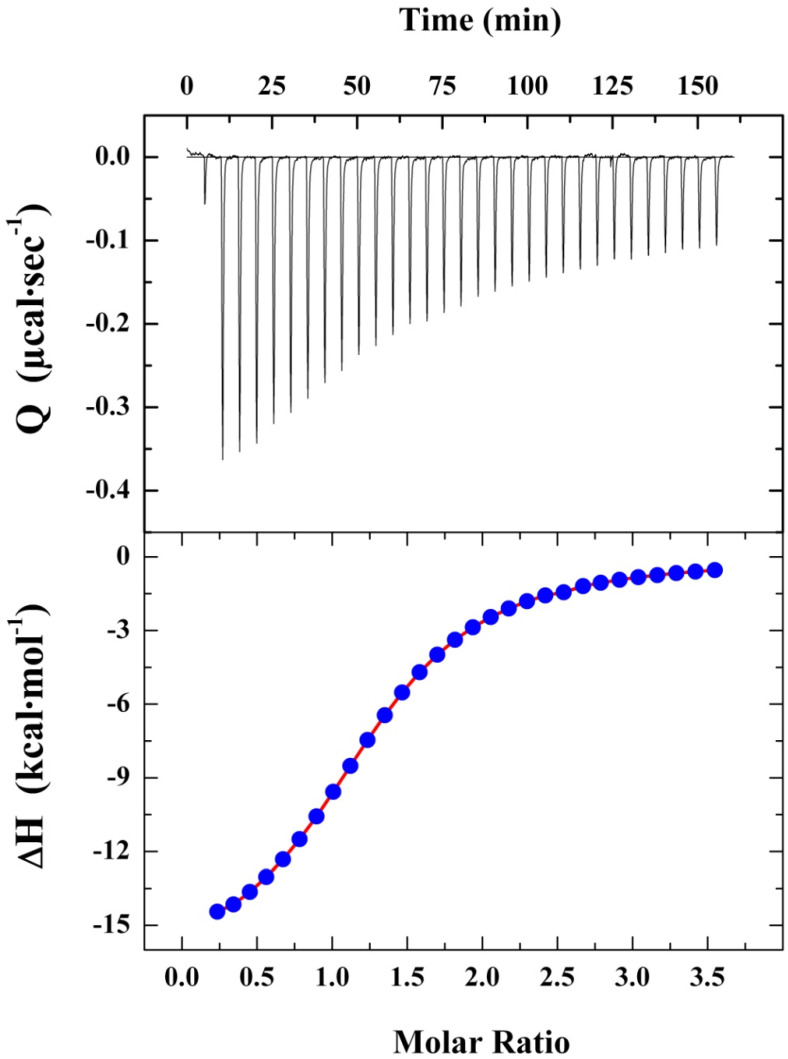
ITC Profile Monitoring Association of the Dichlorohexamer and HSA. The binding isotherm is acquired at 25.0 °C in 10 mM sodium phosphate buffer (pH 7.4). Titration of HSA (5 μM) with the dichlorohexamer (70 μM) is reflected in the thermogram (top panel), integrated peak areas (blue circles), and corresponding fit (red line) to a single site binding model (bottom panel).

Comparable to its interaction with heterogeneous polymeric ATA, the calorimetric profile of HSA binding to a chlorinated hexamer designated as compound **11** (Refer to Scheme 3) is characterized by an enthalpy-driven (ΔH = −15.1 kcal·mol^−1^) process as illustrated in Figure 4. In contrast with the heterogeneous polymeric mixture that is expressed in terms of triphenylmethane units (yielding a stoichiometric ratio of ~4.5), compound **11** is expressed as a single hexameric unit (M_w_ = 956.11). Accordingly, inspection of the resultant ITC profile reveals formation of a 1:1 protein–ligand complex with a single dichlorohexamer occupying an HSA subdomain. Another distinguishing characteristic of the interaction between compound **11** and HSA is its unfavorable entropic nature (TΔS = −7.1 kcal·mol^−1^) which results in a binding free energy (ΔG) of −8.0 kcal·mol^−1^. While HSA association with the chlorinated hexamer and heterogeneous polymeric ATA are characterized by comparable Gibbs Free Energies (ΔΔG = 0.4 kcal·mol^−1^), their thermodynamic binding signatures are remarkably distinct. These results can be rationalized in terms of binding-induced motion restriction within the dichlorohexamer that incurs an entropic penalty. Table 1 presents a summary of thermodynamic binding parameters deduced via analysis of ITC profiles characterizing HSA-ligand interactions for polymeric ATA and the dichlorohexamer.

#### 2.2.4. Interaction of ATA-Derivatives with Host and/or Viral SARS-CoV-2 Targets: A Synthetic Chlorinated Hexamer Binds Ribosomal Particles with Moderate Affinity

**Figure 5:**
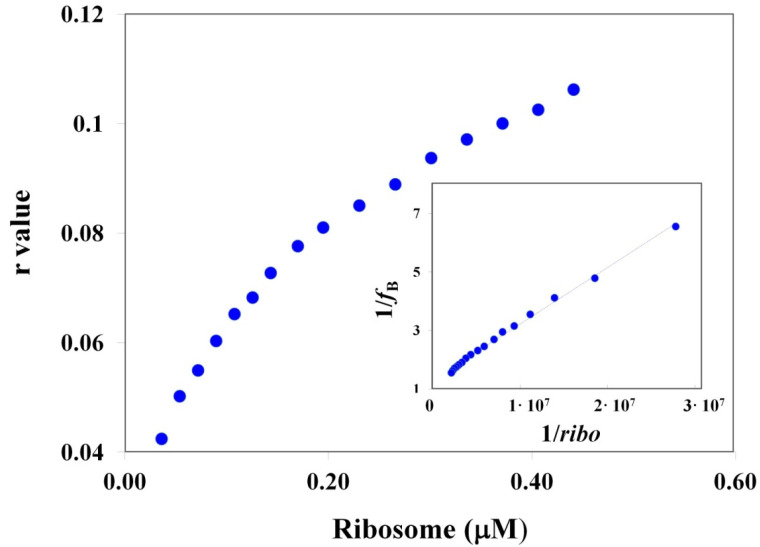
Dichlorohexamer Binding to Yeast Ribosomes Monitored via Fluorescence Anisotropy. Aliquots of purified ribosome stock (27 μM) are titrated into the ligand solution (1 μM) under continuous stirring at 25 °C. Fluorescence anisotropy (r) values monitoring ribosome–ligand binding are measured at excitation/emission wavelengths of 305 nm and 420 nm, respectively. The double reciprocal plot yields an affinity of 4.8 × 10^6^ M^−1^ (K_d_ = 0.21 ± 0.02 μM) for the synthetic chlorinated hexamer **11**.

ATA has been reported to prevent the attachment of bacteriophage messenger RNA to ribosomes [21]. The initiation of protein synthesis in cell-free extracts from *E. coli* or rabbit reticulocytes is inhibited at ATA concentrations (<100 nM) which do not prevent chain extension [23]. We have evaluated the ability of ATA derivatives to interact specifically with yeast 80S ribosomes via fluorescence anisotropy. In this experiment, aliquots of purified ribosome stock are titrated into the ligand solution equilibrated in a quartz cuvette under continuous stirring at 25 °C. Fluorescence anisotropy (r) values monitoring ribosome–ligand binding are measured at excitation/emission wavelengths of 305 nm and 420 nm, respectively. The resultant G-factor-corrected r values are employed to calculate the binding affinities as illustrated in Figure 5. Following analysis of the data via linearized plots as described in Materials and Methods, we measure an affinity of 4.8 × 10 ^6^ M^−1^ (K_d_ = 0.21 ± 0.02 μM) for the synthetic dichlorohexamer.

### 2.3. Biological Properties and Functional Activity

#### 2.3.1. Heterogeneous ATA and a Synthetic Chlorinated Hexamer Inhibit Protein Synthesis in Rabbit Reticulocyte Lysates

**Figure 6:**
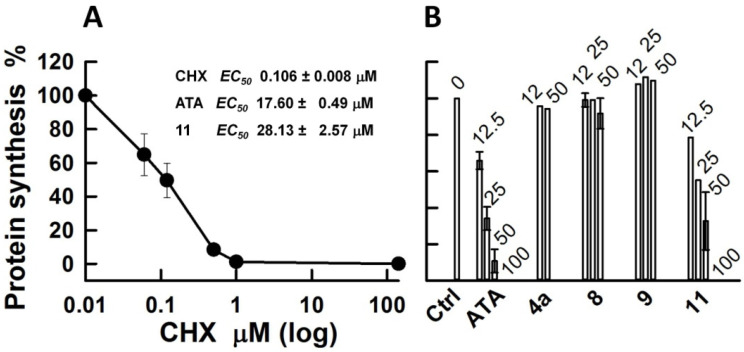
Inhibition of Protein Synthesis by CHX and ATA Derivatives in Rabbit Reticulocyte Lysates. (**A**) Dose-dependent Inhibitory activity of CHX. (**B**) Inhibitory activities of ATA and synthetic derivatives. Concentrations of ATA species are indicated above each bar. Test compounds are incubated with lysates of rabbit reticulocytes in the presence of mRNA coding for luciferase. The chemiluminescence of reaction mixtures is measured following addition of luciferin. Samples incubated in the absence of investigational drugs are used as 100% reference. Results are presented as mean values and standard deviations for three independent measurements; otherwise, data are presented as average values of two independent experiments with 10–15% agreement. EC_50_ values are calculated and displayed in Panel A.

Since ATA has been originally described as an inhibitor of mammalian protein synthesis, we evaluated commercial heterogeneous ATA and synthetic oligomers of various lengths for their potential to affect translation in lysates of rabbit reticulocytes using mRNA coding for firefly luciferase. The dose–response change in chemoluminescense monitored in the presence of various concentrations of investigational drugs following addition of luciferin allows comparison across compounds. Cycloheximide, a known inhibitor of protein synthesis elongation, has been used as a positive control with an EC_50_ (50% inhibitory concentration) of 0.106 μM (Figure 6A). Employing this assay, we analyzed heterogeneous ATA and several intermediates in the synthetic pathway (Refer to Scheme 3). Whereas polymeric ATA exhibits an EC_50_ of 17.6 μM, small molecular weight analogs represented by chlorinated dimer **4a** (a derivative of compound **5** in Scheme 3), chlorinated tetramer (**8**), and dechlorinated tetramer (**9**) did not impact protein synthesis over a concentration range extending to 50 μM. Significantly, the chlorinated hexameric compound (**11**) inhibits protein synthesis with an efficiency (i.e., EC_50_ of 28.13 μM) similar to that of the heterogeneous ATA preparation (Figure 6B). It is worth noting that at 100 μM concentrations, both polymeric ATA and the dichlorohexamer completely inhibited translation, exhibiting a signal comparable to background values (i.e., in the absence of mRNA substrate). These results suggest that a hexamer is the minimum length of ATA-derived species required to inhibit protein synthesis activity.

#### 2.3.2. Heterogeneous ATA and Synthetic Chlorinated Hexamer Inhibit SARS-CoV-2 RNA Dependent RNA Polymerase

**Figure 7:**
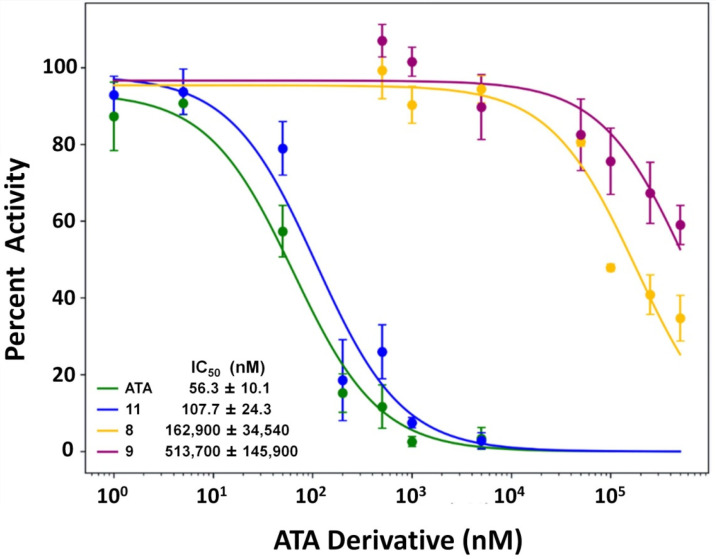
ATA and the Dichlorohexamer Inhibit SARS-CoV-2 RdRp. ATA derivatives are incubated with nsp12/7/8, an active complex of RdRp, in the presence of hybridized RNA substrate and ribonucleoside triphosphates. Cy3-labelled products are resolved via gel electrophoresis and inhibitory activity (IC_50_) of each compound evaluated by fitting the inhibitory curves to a four-parameter logistic regression. Each dose-response curve is generated from three independent experiments. Mean and standard deviation values are plotted for each concentration of test compounds. The full activity (100%) of RdRp in the absence of inhibitors ranged from 33–72 pmol of product depending on the reaction date.

The mechanisms underlying ATA-mediated anti-viral activities consist of early stages in the viral life cycle and conceivably comprise inhibition of viral RNA-dependent RNA polymerase (RdRp). In this study, we assessed the binding and inhibition activities for several host and viral targets including SARS-CoV-2 RdRp. The heterogeneous ATA preparation efficiently inhibits RdRp-mediated RNA replication (IC_50_ = 56 nM), confirming previous assumptions that ATA interacts with and inhibits RdRp. Moreover, amongst the various molecular weight lead compounds evaluated, we find that only the dichlorohexamer retains an ability to inhibit RdRp with a potency comparable to the ATA mixture (IC_50_ = 108 nM). Our data on RdRp polymerase activity reveal that the compounds studied herein rank according to the following order of inhibitory activity: Polymeric ATA (IC_50_ = 56 nM) ~ Dichlorohexamer **11** (IC_50_ = 108 nM) >>> Tetramer **8** (IC_50_ = 163 µM) > Tetramer **9** (IC_50_ = 514 µM). A representative inhibition profile for these compounds is presented in Figure 7. Inspection of the data indicates that a hexamer is the minimum length required for inhibitory activity which is consistent with previous reports that the bioactive species in ATA preparations comprise molecular weights ≥ 1000. These findings suggest that the synthetic dichlorohexamer exhibits an optimal footprint to interact with and inhibit the RdRp polymerase complex at nanomolar potency.

#### 2.3.3. Heterogeneous ATA Is Non-Toxic and Exhibits Limited Activity as a Protein Synthesis Inhibitor in Cultured Vero E6 Cells

**Figure 8:**
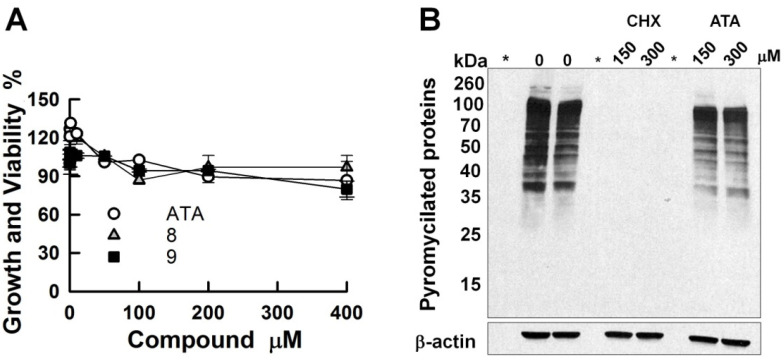
Impact of Heterogeneous ATA and Synthetic Derivatives on Vero E6 Cells in Culture. (**A**) SRB assay following 48 h exposure in culture with ATA, tetramer **8**, and tetramer **9**. (**B**) Representative immunoblottings for puromycilated proteins and ß-actin following 1 h exposure to ATA and cycloheximide (CHX) in culture and puromycin labelling. An asterisk (*) indicates wells that include commercial protein standard. Refer to Figure S5 and Table S1 for additional details.

In an effort to establish the effects of ATA species in cultured cells, we have studied cell growth and viability of a Vero E6 cell line in response to heterogeneous ATA, tetramer **8**, and tetramer **9**. In view of our long-term goal to generate antiviral drug candidates, we selected the Vero E6 cell line that is amenable to infections with various coronaviruses including hCoV-OC43 and SARS-CoV-2. The SRB assay is employed for in vitro cytotoxicity screening and measures the absorbance of total protein proportional to cell biomass. Application of this assay reveals that none of the three compounds affect cell growth and viability at concentrations up to 400 μM over 48 h (Figure 8A). The lack of host cell toxicity is a desirable property for investigational drugs, which usually suggests that there are no mammalian targets for a given compound and antiviral mechanism(s) is/are selective toward the virus. When puromycin labeling is applied to establish whether polymeric ATA inhibits protein synthesis in Vero E6 cells, we observe only a 30–40% response between 150–300 μM ATA, in contrast with CHX, which inhibits protein synthesis at concentrations of 1 μM. Representative data illustrating the differential impacts of ATA and CHX on protein synthesis inhibition are depicted in Figure 8B. The inability of ATA to inhibit protein synthesis in Vero E6 cells might be attributed to the fact that such cells are of renal origin which express P-glycoprotein, a transporter responsible for drug efflux [48]. Another possibility is the charged nature of ATA derivatives, which might prevent passive diffusion into the intracellular space. Since compounds with similar charge exhibit no effects in human lung A549 cells (data not shown) and the latter do not overly express P-glycoprotein, our second hypothesis appears more plausible. Accordingly, other groups have demonstrated that while ATA might inhibit protein synthesis in cell free systems, it may lack this activity in cell culture [49,50]. Our results indicate that further modifications of the carboxyl groups in synthetic ATA species might be required to observe antiviral effects in vitro and in vivo.

## 3. Discussion

Seminal studies on the remarkable antiviral properties of triphenylmethane dyes discovered over four decades ago [21,22,23,25,26] have prompted investigations of their potential use as lead compounds in targeting SARS-CoV-2. In the search for novel activities via HTS assays utilizing the LOPAC arsenal of compounds, ATA is often identified among positive *hits* in drug discovery campaigns, including those in the quest for SARS-CoV-2 therapies [34]. Considering the potential utility of ATA in terms of its multitarget effects, a thorough characterization of biophysical and molecular features including drug-like properties is severely lacking. In view of the intrinsic heterogeneity observed amongst polymeric ATA preparations [35,36,37], there is an urgent need to isolate and/or identify active components for the express purpose of developing and synthesizing lead compounds that retain the characteristic molecular properties. The latter must be achieved while simultaneously ensuring the requisite homogeneity and purity that enables such compounds to be considered as viable drug candidates.

### 3.1. Biophysical Studies Assist Synthetic Efforts in Selecting a Representative Oligomeric ATA Analog

ATA is an anionic polymer that has been demonstrated to bind a variety of viral protein targets including gp120 of HIV-1 and HIV-2 and reported to prevent SARS-CoV replication in cultured cells [33]. A complete understanding of the biological mechanisms underlying antiviral activity necessarily requires a comprehensive multidisciplinary approach to characterize the physicochemical and structural properties of ATA as lead candidate(s). Considering the heterogeneous nature of such compounds, our experimental strategy aims at identifying and evaluating the distribution of oligomeric species/states to assist chemists in the overwhelming task of synthesizing compounds that mimic the bioactive properties of these mixtures yet exhibit well-defined structures and molecular weights.

### 3.2. Characterization of ATA Intrinsic Biophysical Properties

As a prerequisite for studying ATA binding to specific cellular targets involved in SARS-CoV-2 infectivity, we have characterized the optical and calorimetric properties of ATA–HSA interactions. Knowledge of the HSA binding affinity for lead compounds is required to assess the bioavailability of these drug candidates in vivo. Moreover, such studies are critical in terms of advancing/evaluating ADMET properties that are required in drug development. The resultant data provide unique insights into specific binding modes, stoichiometry, and driving forces underlying ATA–HSA interactions. Our biophysical approach for characterizing ATA binding properties to the ubiquitous protein carrier/receptor HSA has proven particularly insightful.

Employing a combination of optical and calorimetric techniques, we have defined the primary ATA polymeric binding species and determined the requisite binding affinity, stoichiometry, and thermodynamic signatures to dissect the enthalpic and entropic components driving protein–ligand association. Characterization of the ATA–HSA thermodynamic profiles serves as a reference for elucidating specific interactions with other competing cellular targets, including SARS-CoV-2 RdRp and/or the ribosomal site(s) where viral protein synthesis initiation occurs. Collectively, these studies map the binding profiles and thermodynamic signatures of drug–target and off-target interactions that are related to their antiviral biological activities and thereby provide an additional layer of information for rational drug design/optimization, decision-making protocols during screenings, and structure–energetic activities in antiviral drug discovery.

This study provides the basis for synthesis of a lead compound resembling the polymeric ATA mixture that has been characterized in terms of its structural identity and homogeneity via mass spectrometric and NMR analysis in conjunction with concentration-dependent optical and calorimetric studies. Our data corroborate a wealth of published studies in which the ATA bioactive species is comprised of a polymeric mixture *in lieu of* well-defined monomeric structures. Following confirmation of the identity, purity, and molecular weight, we have characterized the binding energetics of this bioactive compound with an off-target transporter protein (HSA) and its respective viral target(s). Specifically, we have identified structural features of ATA-derived compounds that drive the observed antiviral activity.

### 3.3. Binding Profiles of Polymeric ATA versus the Synthetic Dichlorohexamer

ATA is suggested to adopt polymeric structures that are able to interact with and inhibit the helicase from hepatitis C NS3 virus [51]. However, unlike the branched structure proposed by Gonzalez et al. [36], the authors contend that ATA adopts a more linear polymeric arrangement which mimics nucleic acid structures [51,52]. Inspection of the ITC profile for ATA binding to HSA (Figure 3) reveals an average stoichiometry of ~5 ATA monomeric units per protein, a finding that infers the predominant molecular weight species is on the order of 2000 Da, which is consistent with fractionation studies conducted previously [36]. Considering the idealized structure proposed by Cushman et al. [35] and our observation that the active ATA component is comprised of approximately five monomeric units, we have designed an experimental strategy to synthesize linear oligomeric compounds with the goal of identifying an optimal molecular weight and length that exhibits maximal activity.

Pursuing this synthetic strategy, we have isolated a dichlorohexamer that interacts with HSA via a single site binding mode and exhibits a stoichiometry of one hexamer per HSA molecule (Figure 4). Comparison of the respective HSA binding profiles for polymeric ATA and the dichlorohexamer reveals comparable Gibbs free energies yet their thermodynamic signatures are quite distinct (Table 1) as the ATA analog exhibits both a higher enthalpy and unfavorable entropy. While ITC studies on the viral targets are deferred to a subsequent study, we can infer that the dichlorohexamer retains biophysical properties commensurate with desirable drug-like qualities such as the hydrophilicity required for solubility and bioavailability. Moreover, as a lead compound that exhibits a high enthalpic efficiency, the ATA analog may outperform prospective competitors that are predominantly entropy-driven with concomitant insolubility and loss of target selectivity [53].

Enthalpic efficiency is considered a unique advantage in drug discovery [53,54] as this metric is proposed to confer higher target selectivity while optimizing ADMET properties (as reviewed in [55]). The enthalpic nature of dichlorohexamer–HSA interactions is consistent with the polarity of prospective lead compounds and suggests that this ATA analog may exhibit the requisite specificity of an antiviral drug provided interactions with specific targets are comparable to those observed for HSA binding. Studies are currently in progress to evaluate this hypothesis by characterizing the thermodynamics of ATA derivative interactions with various host and viral targets. The chlorinated hexamer has been compared with polymeric ATA in terms of its biological and biomolecular properties, revealing similar or superior potency relative to commercially available ATA preparations as described in the following sections.

### 3.4. ATA Derivatives Bind and Inhibit SARS-CoV-2 Targets

Antiviral drugs exhibit a broad range of mechanisms to target one or more steps in the virus life cycle within mammalian cells. These include blocking cell entry, inhibiting viral proteases required for polypeptide cleavage yielding active viral proteins, and preventing interactions between the virus and host cell that are crucial for viral reproduction. Specific drugs inhibit various components that are exploited by the virus for protein synthesis (e.g., the mammalian ribosome) and replication of its genetic material (i.e., viral RNA-dependent RNA polymerase). This study focuses on host ribosomal protein synthesis machinery and SARS-CoV-2 RdRp. Recent findings suggest that ATA exhibits antiviral activities against SARS-CoV [33] and SARS-CoV-2 [34,56] which reinforces our strategy to identify the antiviral bioactive species in ATA preparations and characterize their respective target(s).

### 3.5. ATA Derivatives Bind Host Ribosomes and Inhibit Protein Synthesis Initiation

Early studies [21,31,57] reported on ATA inhibition of messenger RNA attachment to ribosomes with negligible effect on aminoacyl transfer to peptide. The initiation of protein synthesis in rabbit reticulocytes is impacted by ATA at concentrations (<100 nM) which do not prevent chain extension [21,58]. The remarkable properties of ATA inhibiting ribonucleic acid attachment to the ribosome and halting protein synthesis initiation has sparked broader interest in exploring its prospective role as an effective antiviral agent and prompted us to investigate the binding properties and inhibition mechanisms of ATA derivatives towards eukaryotic ribosomes. Inspection of fluorescence anisotropy binding profiles (Figure 5) reveal that the dichlorohexamer exhibits a preferential affinity for purified yeast ribosomes (i.e., K_d_~200 nM) nearly ten-fold higher than its association with the non-target HSA.

In an effort to explore the underlying mechanisms triggered by specific interactions with purified ribosomes, we have assessed the impact of various ATA-derived compounds on protein synthesis by reticulocyte lysate ribosomes (Figure 6). Analysis of the resultant data reveals that these compounds inhibit protein synthesis in reticulocytes thereby corroborating prior studies. In contrast with equilibrium binding assays, the IC_50_s for protein synthesis inhibition in reticulocytes are substantially higher (on the order of 20–30 μM). At concentrations of 100 μM, both ATA and the dichlorohexamer inhibit translation by nearly 100%. While the origin of these results requires further exploration, the data suggest that there are a number of concurrent events (i.e., on target/off target) in the reticulocyte lysate ribosome samples. These might recruit ATA and its analog which compete with protein synthesis inhibitory mechanisms and thereby require higher concentrations for effective protein synthesis inhibition. Our ongoing studies are aimed at identifying the origins of these intriguing results.

### 3.6. ATA Derivatives Bind and Inhibit SARS-CoV-2 RdRp

Direct ATA-target binding interactions have been reported for a number of nucleic acid processing enzymes including DNase I (K_d_ = 9 μM), RNase A (K_d_ = 2.3 μM), reverse transcriptase (K_d_ = 0.25 μM), and Taq polymerase (K_d_ = 82 μM) as measured via fluorescence studies [59]. The overall mechanisms ascribed to ATA derivatives reside in the ability to inhibit crucial macromolecular processes via substrate/cofactor displacement and/or competition mechanisms in addition to direct interaction with the target enzymes. Examples of ATA-mediated inhibition processes that likely displace their natural substrate(s) include several polymerases, helicases, and other viral enzyme targets [29,52,60,61,62]. The fact that ATA is efficacious against a number of viruses [26] including coronaviruses and a potential mechanism of action is viral replication inhibition [25], a logical strategy is to assess the impact of such compounds on SARS-CoV-2 RdRp.

The current study reports direct inhibition of SARS-CoV-2 RdRp by polymeric ATA and synthetic oligomers with nanomolar potencies. Amongst several derivatives studied, the dichlorohexamer is nearly equipotent (IC_50_ = 108 nM) to heterogeneous ATA (IC_50_ = 58 nM) in inhibiting the RdRp-dependent RNA replication complex (Figure 7). Comparing these results with data on other nucleic acid enzymes (i.e., K_d_/IC_50_ ranging from 0.25 to 82 μM) [59], and assuming the interchangeability of K_d_ and IC_50_ (an approximation that is not always warranted), the dichlorohexamer represents a high affinity candidate with an IC_50_ ranking among some of the highest potency inhibitors. In fact, recent studies report RdRp inhibitors with IC_50_s in the low micromolar range, values that are comparable to those observed for remdesivir as a control compound [63].

### 3.7. ATA Analog Bioactive Conformation as RdRp Inhibitor

Despite the wealth of experimental evidence accumulated to date that confirms the heterogeneous polymeric nature of ATA preparations, computational models with docking poses suggest that the ATA monomer (Scheme 1) binds SARS-CoV RdRp at the target site(s). Our experimental strategy employing a combination of synthetic, biological, and biophysical protocols has confirmed a minimal length for effective inhibitor activity. Specifically, the dichlorohexamer retains comparable activity to the polymeric ATA mixture, whereas tetrameric compounds **8** and **9** exhibit minimal or no RdRp inhibition activity (i.e., IC_50_ ~ 163 and 514 μM, respectively) as illustrated in Figure 7. Efforts towards identifying suitable lead compounds with proven antiviral activity and preferably targeting RdRp should provide the basis for developing effective therapeutics against SARS-CoV-2 and facilitating the design of broad-spectrum antivirals that may be useful in combating future coronavirus outbreaks. This study describes the characterization of ATA-derived compounds selected on the basis of their homogeneity, binding, inhibition, and antiviral activities, essential characteristics that serve as a lead for the generation of novel compounds with a clear therapeutic efficacy.

### 3.8. ATA and Derivatives in the Cellular Context: Permeability and Toxicity

Although antiviral activity has been demonstrated for ATA against SARS-CoV in the absence of toxicity to host cells [33], the latter is achieved only at significantly high concentrations (i.e., 200–500 μM). Recently, it has been reported that ATA inhibits propagation of SARS-CoV-2 in VERO cells [56] albeit at concentrations as high as 100 μM to achieve full antiviral activity. Our findings are generally consistent, as we observe modest activity when assessing the antiviral properties of ATA and dichlorohexamer at concentrations ≥200 μM (data not shown) in hCoV-OC43 and VERO E6 cells. Given the inhibitory activity of ATA towards mammalian protein synthesis in cell-free systems and the lack of toxicity in host cells, we hypothesize that ATA antiviral activity generally occurs at high doses and is elicited via inhibition of viral entry. In fact, investigators have identified another mechanism by which ATA may exert antiviral activity that involves inhibition of cell-surface receptor (i.e., acetyl cholinesterase 2) binding by the RBD (receptor binding domain) of the SARS-CoV-2 spike protein [34]. While a recent study claims that ATA is membrane-permeable in a rat brain PC12 cell line, the concentrations required to monitor its accumulation within cells is on the order of 300 μM [64]. The unusual choice of host cells selected for these measurements warrants caution in interpreting their results.

In view of these disparate findings and our experimental observations, we contend that neither ATA nor the isolated synthetic derivatives retain an ability to readily penetrate cultured cells. Moreover, the ATA species that affect viral entry are conceivably distinct from those inhibiting viral RdRp and/or protein synthesis, which require resolution of ATA components within heterogeneous preparations. The most plausible explanation for a lack of cell permeability is that the charged nature of ATA compounds precludes passive diffusion into the intracellular space, thereby exhibiting minimal or no activity in cultured cells. In an effort to address this deficiency, our ongoing studies are aimed at optimizing ATA-derived lead compounds in order to preserve potency while simultaneously improving their ability to enter mammalian cells. As an alternate approach, specific ATA analog delivery systems will be evaluated in terms of the ability to facilitate cell entry/permeability and thereby exert their full potential as antiviral molecules.

### 3.9. Structure-Activity Considerations regarding ATA Derived Lead Compounds

**Figure 9:**
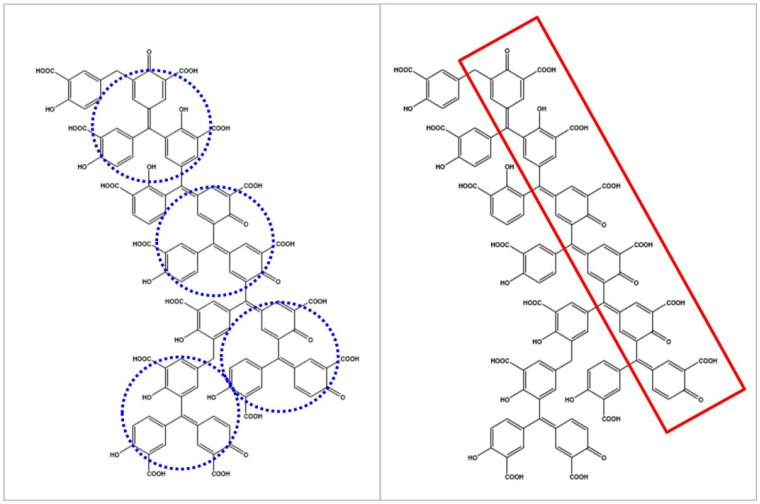
An Idealized Binding Mode Based on Biological and Biophysical Properties. Considering the structure proposed by Cushman et al. [35] depicting triphenylmethane units in blue (**left** panel) and molecular weight deduced from the ITC stoichiometric ratio in conjunction with inhibition of RdRp exhibited by the dichlorohexamer, we envision that the polysalicylic acid stretch outlined in red (**right** panel) corresponds to a minimal optimal enzyme footprint for ATA activity. Reduction of the quinomethine groups yielding methylsalicylic acid polymers such as the dichlorohexamer does not abolish the activity of these ATA analogs.

Although officially reported as triphenylmethane (CID: 2259; M_W_ = 422.34 g/mol), ATA in fact comprises a heterogeneous polymeric population of various molecular weight species. Investigations utilizing ATA in screenings generally assume with rare exception that this compound exists in the monomeric state which is employed for in silico docking. Unlike numerous studies that persist in perpetuating the “active” ATA species as a monomer, a recent study suggested that ATA indeed adopts polymeric structures and the latter are effective helicase inhibitors [51]. These authors have correctly suggested that ATA adopts a more linear polymeric arrangement resembling nucleic acid structures similar to the proposal of Cushman and colleagues [52]. Our combined biological/biochemical/biophysical data characterizing the ATA-elicited RdRp inhibition properties suggest that a minimum optimal oligomeric size is required for viral activity. Considering the proposed idealized structure [35] and controlled synthesis of linear oligomeric compounds presented herein, we envision specific interactions of the ATA-derived polymers to occur via a polysalicylic stretch linearly distributed as illustrated in Figure 9, which may resemble nucleic acid substrates. While the dichlorohexamer mimics or even exceeds the biological properties of heterogeneous ATA, we are expanding our synthetic repertoire to generate higher molecular weight structures (e.g., an octamer) that are the focus of a forthcoming study.

## 4. Concluding Remarks

Given the recent emergence of SARS-CoV-2 as a global pandemic, coupled with the need to maintain vigilance in a post-pandemic/endemic era, the identification of potent antiviral therapies represents an urgent priority with the surge of novel variants exhibiting resistance to current vaccine regimens. Recent studies screening thousands of novel and repurposed compounds have identified ATA as a prospective lead candidate in SARS-CoV-2 treatment strategies [34,56,65,66,67]. ATA preparations currently employed for screening purposes comprise a heterogeneous mixture of active/inactive components that preclude drug development and lead optimization. The current study represents an inaugural attempt to alleviate such shortcomings by synthesizing potent homogeneous ATA derivatives harboring specific therapeutic properties. The latter must meet or exceed that of the active heterogeneous ATA preparations, thereby serving the purpose of identifying effective anti-SARS-CoV-2 agents with potential broad spectrum therapeutic applications in the drug discovery arena. The finding that a hexameric salicylic acid derivative exhibits optimal SARS-CoV-2 RdRp inhibition activity underscores the potential viability of these compounds as lead candidates in antiviral treatment therapies and remains the focus of our ongoing studies.

## 5. Materials and Methods

### 5.1. Materials

Aurintricarboxylic Acid (ATA) was procured from Millipore Sigma (St. Louis, MO, USA) as catalog number A1895 with a designated M_w_ of 422.34. ATA was suspended without further purification/treatment in 10 mM sodium phosphate buffer (pH 7.4) or dialysate for calorimetric characterization of protein-ligand binding interactions. Defatted Human Serum Albumin (HSA) was obtained from Millipore Sigma (St. Louis, MO, USA) as catalog number A1887. HSA stock solutions (100 µM) were prepared by suspending the lyophilized powder in 10 mM phosphate buffer containing 1 mM mercaptoethanol (BME) to ensure disruption of Cys-34 crosslinked dimers. The HSA solution was dialyzed extensively against three 1.0 L exchanges of 10 mM sodium phosphate buffer (pH 7.4) to remove excess BME and the resultant monomeric mercaptoalbumin used for biophysical measurements. The HSA standard concentration was determined spectrophotometrically employing an extinction coefficient of 35,000 M^−1^ cm^−1^ at 280 nm. Cycloheximide (CHX), TWEEN20, sodium dodecyl sulfate (SDS), trypsin-EDTA, TRIS-buffered saline, Dulbecco’s phosphate-buffered saline (PBS), methanol, puromycin, and film for chemoluminescence detection were obtained from Millipore Sigma (St. Louis, MO, USA). Unless indicated otherwise, reagents, materials and equipment for electrophoresis and immunobloting were purchased from Bio-Rad Laboratories (Hercules, CA, USA). The sources of other relevant reagents, biological kits, and materials are noted in the protocols described below.

### 5.2. UV/Vis Absorbance and Fluorescence Spectroscopy

#### 5.2.1. Characterization of HSA and ATA Optical Properties

The optical properties of HSA, heterogeneous polymeric ATA, and oligomeric derivatives have been characterized by monitoring their respective absorbance profiles on an AVIV Biomedical Model 14 UV/Vis Spectrophotometer (Lakewood, NJ, USA). Absorbance spectra have been acquired at 1.0 nm intervals over the wavelength range of 200–800 nm employing an averaging time of 5 s and slit width of 1 nm. The profiles are characterized by absorbance peaks centered at 280 nm and 310 nm for HSA and ATA, respectively. In view of the fact that drugs tend to aggregate as a consequence of their intrinsic hydrophobicities, it is important to assess the state of these ligands in terms of biophysical and structural properties. Concentration-dependent measurements of polymeric ATA standards spanning the 10 to 2500 µM range have been conducted to determine its propensity for intramolecular aggregation. Expanding these measurements to the nanomolar range, concentration-dependent studies on the fluorimetric properties of polymeric ATA standards ranging from 10 to 1000 nM have been evaluated. Analysis of the respective absorbance and fluorescence spectra reveals a strictly linear dependence of optical density and emission intensity over this expanded concentration range. These findings suggest that polymeric ATA does not exhibit appreciable intermolecular aggregation and/or form supramolecular assemblies.

#### 5.2.2. Characterization of HSA and ATA Fluorescence Properties

The fluorescent properties of HSA, heterogeneous polymeric ATA, and oligomeric derivatives have been characterized by monitoring their respective profiles on an AVIV Biomedical Model 107 Differential Fluorescence Spectrophotometer (Lakewood, NJ, USA). Fluorescence emission spectra of HSA and ATA analogs have been acquired in a 10 mm quartz cuvette at 1.0 nm intervals over the wavelength range of 250–600 nm employing an averaging time of 5 s and excitation/emission slits of 10 nm. Excitation of an HSA standard at 280 nm yields an emission spectrum with a maximum intensity at 320 nm, whereas excitation of a polymeric ATA standard at 310 nm yields an emission spectrum with a maximum intensity at 425 nm.

#### 5.2.3. Characterization of ATA–HSA Binding via Fluorescence Quenching

Fluorescence quenching measurements of protein–ligand binding interactions monitor the emission intensity of aromatic chromophores including Tyr and Trp residues at 280 and 295 nm, respectively. The impact of polymeric ATA on HSA fluorescent properties has been evaluated via measurement of the protein emission intensity in the presence of increasing ligand. The ATA–HSA interaction has been characterized by titrating aliquots of ligand stock into the protein standard in 10 mM phosphate buffer (pH 7.4) and monitoring the resultant fluorescence emission at 325 nm employing an excitation wavelength of 280 nm. The HSA fluorescence quenching data monitored at 325 nm as a function of increasing ATA concentrations (Figure 2) are recast in the form of a Stern-Volmer plot and analyzed in accordance with the following relation: *F*_0_/*F* = 1 + *K_SV_* [*ATA*]. The ATA–HSA binding profile has also been analyzed via a double-reciprocal logarithmic plot according to the relation: *log* [*F*_0_ − *F*/*F*] = *n log K_b_* − *n log* [1/[*Q*] − (*F*_0_ − *F*) × [*P_t_*]/*F*_0_] where: *Q* = ATA concentration; *P_t_* = total HSA concentration; *F*_0_ and *F* are HSA fluorescence in the absence and presence of ATA. Employing a reverse titration mode monitoring the addition of HSA into ATA, one observes a significant increase in ATA emission intensity at 425 nm upon excitation at 310 nm, suggesting the existence of protein-induced enhancement of ligand fluorescence. This phenomenon is interpreted as the result of enhanced ligand fluorescence upon migrating from an aqueous to protein site environment and corroborates the existence of a specific binding interaction.

#### 5.2.4. Characterization of Ligand–Ribosome Binding via Fluorescence Anisotropy

Fluorescence anisotropy (*r*) of ribosome–ligand binding interactions are performed by exciting the ligand fluorophore (ATA and/or derivatives) with polarized light (310 nm) and measuring the fluorescence intensities (420 nm) that are both parallel (*I_VV_*) and perpendicular (*I_VH_*) to the excitation polarization. The resultant values of *I_VV_*, *I_VH_*, and grating factor (*G*) are introduced into the following relation to calculate fluorescence anisotropy: *r* *= I_VV_* *− G·I_VH_/I_VV_* + 2*G* *× I_VH_*. The *G* value corrects for efficiency differences in the instrument optics [68] and is determined by measuring the intensity ratio of vertical and horizontal components when the free fluorescent ligand solution is excited with horizontally polarized light. Fluorescence anisotropy measurements are performed in 1.0 cm quartz cuvettes under continuous stirring at 25 °C in the presence of increasing ribosome concentrations until saturation is achieved. The interaction of ATA derivatives with ribosomes is characterized by titrating 1–2 μL aliquots of ribosome stock (27 µM) into the ligand solutions in 20 mM PIPES (pH 7.4), 5 mM MgCl_2_, 30 mM KCl, and measuring the r values for calculation of fraction bound (*f_B_*). The latter is determined via the following relation: *f_B_* *= r* *− r_F_/R* × (*r_B_* *− r*) *+ r* *− r_F_*, where *r* is the anisotropy measured at a given ribosome concentration with *r_F_* and *r_B_* corresponding to the anisotropy of free and fully bound ligand, respectively. Since the ligand fluorescence intensity increases in the presence of a targets as observed for ATA–HSA interactions (refer to Figures S1 and S2), a correction factor (*R*) is introduced in the equation to account for these changes [69]. Parallel fluorescence intensity measurements are performed to determine the *R* value which is calculated as follows: *R = F_B_/F_F_*, where *F_B_* and *F_F_* represent ligand fluorescence intensities in the bound and free states, respectively. Recasting *f_B_* as a function of ribosome concentration via a double-reciprocal plot yields the binding constant (*K_b_*) according to the relation: *1/f_B_* = 1/*K_b_* × 1/[*ribosome*] + 1.

### 5.3. Characterization of Binding Energetics via Isothermal Titration Calorimetry

Thermodynamic binding parameters for the association of polymeric ATA and oligomeric derivatives with Human Serum Albumin (HSA) were determined calorimetrically employing a MicroCal VP-ITC (Malvern Panalytical, Northampton, MA, USA). The protein was prepared as described in Section 5.1 and dialyzed exhaustively against a buffer comprised of 10 mM sodium phosphate adjusted to pH 7.4. The ATA polymer and dichlorohexamer were dissolved directly in final dialysate at the respective standard concentrations. Each ITC experiment consisted of thirty consecutive 10.0 µL injections during which the reaction heat is monitored and integrated over a 5.0 min period under continuous stirring. The experimental protocol has been designed to ensure that there is a tenfold excess of ligand in the titration syringe relative to standard protein concentration in the sample cell. The resultant binding isotherms are generated by recording the integrated reaction heats normalized for ligand concentration versus the HSA:ligand ratio. A nonlinear least squares fit of the resultant profile to a single site binding model yields thermodynamic parameters for the HSA–ligand complex including the affinity (*K_a_*), Gibbs free energy (Δ*G*), enthalpy (Δ*H*), and entropy (Δ*S*).

### 5.4. Biochemical and Biological Assays

The following solutions of ATA analogs were prepared for biological studies. Stock solutions of synthetic ATA species (compounds **8**, **9**, and **11**) were prepared in DMSO at 50–100 mM and stored at −80 °C. Commercial heterogeneous polymeric ATA (catalog number A1895) was obtained from Millipore Sigma (St. Louis, MO, USA). A stock solution containing 15.8 mg/mL was prepared in DMSO and labeled 37 mM ATA employing the molecular weight of 422.3 for a monomeric ATA unit.

#### 5.4.1. Inhibition of SARS-CoV-2 RNA Dependent RNA Polymerase Active Complex

Non-structural proteins 12, 7, and 8 (Nsp12, 7, and 8) of SARS-CoV-2 were purified as described previously [70]. The RNA extension assay was performed in a reaction buffer containing 50 mM KCl, 100 mM Tris-HCl (pH 8.0) and 1 mM DTT. A fluorescent-labeled RNA primer (5′-Cy3-Gr Ur Cr Ar Ur Ur Cr Ur Cr Cr Ur Ar Ar Gr Ar Ar Gr Cr Ur Ar-3′) was annealed to a 40 nt RNA template (5′-Cr Ur Ar Ur Cr Cr Cr Cr Ar Ur Gr Ur Gr Ar Ur Ur Ur Ur Ar Ar Ur Ar Gr Cr Ur Ur Cr Ur Ur Ar Gr Gr Ar Gr Ar Ar Ur Gr Ar Cr-3′) by heating the reaction to 75 °C and gradually cooling to 4 °C which generated a double-strand RNA substrate. SARS-CoV-2 RdRp (nsp12) was incubated with its co-factors nsp7 and nsp8 (1:2:2 molar ratio) on ice for 20 min prior to polymerase extension. The reactions (20 μL) contained 15 mM MgCl_2_, 500 nM RNA substrate, 500 μM NTPs, varying concentrations of ATA species and were initiated by the addition of 1 μM pre-incubated RdRp complex at 37 °C. The reactions were terminated after 60 min by adding 10 μL of stop solution comprised of 95% formamide in 20 mM EDTA. A 5 μL aliquot of reaction product was loaded onto a 16% denaturing polyacrylamide gel and the Cy3-labelled RNA products visualized using a Typhoon Imager. Experiments on each ATA analog were repeated independently at least three times.

#### 5.4.2. Inhibition of Protein Synthesis in Rabbit Reticulocyte Lysates

The TnT^®^ Quick Coupled Transcription/Translation System from Promega (Madison, WI, USA; L1170) was used to evaluate the inhibitory effects of ATA derivatives and cycloheximide on protein synthesis. Assay conditions were employed as recommended by the manufacturer. In summary, reactions (20 μL) contained 80% TNT Quick master mix, 20 µM methionine, and 40 ng/µL luciferase RNA (Promega; L4561) at the indicated concentration of test chemicals. Samples were incubated at 30 °C for 90 min and diluted ten-fold with PBS. An aliquot (2 μL) was mixed with 20 μL of luciferase assay reagent (Promega; E1500) and luminescence was measured with a Turner Designs Model TD-20/20 Luminometer. Depending on the compound evaluated, experiments were repeated two-four times with good agreement between the runs. For compounds that exhibited minimal or no activity, two independent experiments were conducted to confirm the findings. The results are reported as average values including the mean and standard deviation wherever applicable.

#### 5.4.3. Cell Culture

African green monkey kidney cells (Vero E6) were purchased from the American Type Culture Collection (Manassas, VA, USA; Cat. No. VERO C1008 (Vero 76, clone E6, Vero E6) CRL-1586^TM^). Complete details regarding this cell line may be accessed at the following link: https://www.atcc.org/products/crl-1586#detailed-product-information (accessed on 24 April 2022). Prior to commercial availability, the cell lines are tested by the vendor for mycoplasma contamination and authenticated via short tandem repeat profiling. The Vero E6 cell line was handled and maintained according to manufacturer’s instructions in Eagles’s Minimum Essential Medium (EMEM) supplemented with 10% fetal bovine serum (10% FBS-EMEM) in a humidified incubator in 5% CO_2_ at 37 °C.

#### 5.4.4. Protein Synthesis in Cultured Cells: Puromycin Pulse Labeling

Prior to the experiment, Vero E6 cells were seeded at 30,000 cells per cm^2^ in 6-well plates in 5% FBS-EMEM. Two days following plating, the medium was replaced with 5% FBS–EMEM containing polymeric ATA at concentrations spanning the range of 1–300 μM and the plates incubated for one hour followed by addition of puromycin (0.01 mg/mL final concentration). Control experiments were conducted in the absence and presence of cycloheximide (1–150 μM) or DMSO. Following incubation with puromycin for 10 min, the medium was quickly removed and cells washed with warm PBS and allowed to recover in drug-free 5% FBS-EMEM for 30 min. Cell pellets collected by trypsinization and centrifugation were stored at −80 °C while awaiting protein preparation and immunoblotting as described below.

#### 5.4.5. Protein Preparation and Immunoblotting to Detect Puromycilated Proteins

Cell lysates were prepared in RIPA (radio-immunoprecipitation) buffer (Sigma; R0278) containing protease inhibitors (Sigma; P8340). Protein concentrations were determined via the Pierce^TM^ bicinconinic acid (BCA) assay (Thermo Fisher Scientific, Waltham, MA, USA) using bovine serum albumin as a standard. All procedures were performed in accordance with protocols recommended by the manufacturers. For immunoblotting, 8 μg of protein was combined with Laemmli buffer containing 10% β-mercaptoethanol, heated at 95 °C for 5 min and loaded on 8–16% Mini-PROTEAN^®^ TGX™ precast protein gels. Samples were resolved via electrophoresis in Tris-Gly-SDS buffer and transferred to nitrocellulose membranes in Tris-Gly buffer containing 20% methanol at 4–6 V/cm^2^ for one hour. Membranes were blocked in 10 mM sodium phosphate buffer containing 150 mM NaCl (pH 7.8), 5% milk and 0.1% TWEEN20 (PBST) at room temperature for at least one hour, then incubated overnight with primary anti-puromycin antibodies (TFS; MABE343 mouse monoclonal antibodies clone 12D10; 1:25,000) at 4 °C. Membranes were washed three times for five minutes with PBST and incubated with secondary goat anti-mouse horseradish peroxidase (HRP)-conjugated antibodies (TFS, Invitrogen; G21040; 1:25,000) for 90 min. Following three additional rounds of washing as described above, membranes were incubated with an HRP substrate (TFS; 34,077) for one minute and chemoluminescence visualized by exposing membranes to film for various times.

For β-actin staining, the same membranes used for puromycin immunostaining were washed four times for five min with 20 mM Tris-buffered saline containing 500 mM sodium chloride (pH 7.5) and 0.1% TWEEN20 (TBST) and stripped by two rounds of 20- and 10-min washes at 50 °C in glycine buffer (pH 2.2) containing 0.1% SDS (*w/v*) and 1% TWEEN20. Membranes were then washed at room temperature with PBS (3 × 10 min) and PBST (2 × 5 min). Blocking, primary (1:4000), and secondary (1:5000) antibody staining were conducted as described above. Primary rabbit anti-β-actin antibodies (4967S, lot 9) and secondary HRP-conjugated goat-anti-rabbit antibodies (7074S, lot 27) were obtained from Cell Signaling. The results were quantified with Image J 1.52a software using β-actin staining for each sample as an internal reference. A second set of puromycin labeling experiments was conducted to confirm the findings and results are displayed as a representative immunoblotting.

#### 5.4.6. Evaluation of Growth Inhibition and Toxicity in Vero E6 Cells

A day before exposure, Vero E6 cells were plated on 24-well plates (500 μL per well) at 6250 cells per cm^2^ in 10% FBS-EMEM. Test compounds were dissolved in EMEM at two-fold concentrations from their designated amounts in culture and added to cells in equal volume (500 μL) to achieve concentrations of 1–400 μM in 5% FBS-EMEM. The cells were exposed under standard culture conditions for 48 h. Controls containing varying amounts of DMSO and sans DMSO were included as well. A sulforhodamine B (SRB) assay was conducted to evaluate the impact of polymeric ATA and oligomeric analogs on cell growth and survival. All procedures were conducted according to protocols employed by the National Cancer Institute that are available online at https://dtp.cancer.gov/discovery_development/nci-60/methodology.htm (accessed on 24 April 2022). In summary, the exposed cells were fixed by addition of trichloroacetic acid (TCA, 10% *v/v*) and incubated for one hour at 4 °C after which the plates were washed five times with tap water and air dried. For color development, 500 μL of 0.4% SRB reagent prepared in 1% acetic acid was added to fixed cells and the samples were incubated at room temperature for 15 min under gentle rocking. Unbound dye was removed via five washes in 1% acetic acid and the plates air dried. Trizma base (10 mM) was used to dissolve the stain and absorbances recorded at 515 nm on a UV/Vis Model Ultrospec2000 Spectrophotometer (PharmaciaBiotech, Piscataway, NJ, USA). Since DMSO did not affect cells at the concentrations employed in our assay, the absorbance values of cells incubated in media with or without DMSO for 48 h were combined and established as 100% growth/survival. We also conducted an SRB assay on separate wells of cells the same day of exposure (day 0) taking advantage of the ability to store fixed plates for parallel processing with samples following exposure. All experiments were repeated two/three times independently, two/three wells per each condition within an experiment. Dose-dependent changes in survival and cell growth were analyzed via the Sigma Plot Program v.13.0 (Systat Software, Inc., San Jose, CA, USA) and displayed as mean values with standard deviations.

#### 5.4.7. Isolation of 80S Yeast Ribosomes

*Saccharomyces cerevisiae* YPL 154C strain (MATa leu2Δ0 met15Δ0 ura3Δ0 pep4Δ::kan^r^, Horizon Discovery, Lafayette, CO, USA) was grown in YPD medium and disrupted in liquid nitrogen by SPEX 6870 freezer mill. Yeast cell extracts were prepared in a buffer system comprised of 50 mM Hepes-KOH (pH 7.4), 11 mM magnesium acetate, 1 mM dithiothreitol, 1 mM phenylmethylsulfonyl fluoride, cOmplete Protease Inhibitor Cocktail (1 tablet/50 mL, Roche 11697498001), and 60 U/mL Protector RNase Inhibitor (Roche 03335402001). The supernatant was obtained via ultracentrifugation in a SW32Ti rotor (Beckman, Brea, CA, USA) at 13,000 rpm (max 30,000× *g*) for 45 m at 4 °C. The supernatant was loaded onto 37.65% (*w/v*) sucrose, 20 mM Bis-Tris (pH 6.5), 11 mM magnesium acetate, 0.2 M ammonium acetate, 0.3 M potassium chloride and 5 mM dithiothreitol. Ultracentrifugation was performed in a SW32Ti rotor (Beckman) at 28,000 rpm (max 140,000× *g*) for 22 h at 4 °C. The supernatant was decanted and the pellet washed twice with a buffer composed of 20 mM Hepes-KOH (pH 7.4), 11 mM magnesium acetate, 30 mM potassium chloride, 2 mM dithiothreitol, cOmplete Protease Inhibitor Cocktail (1 tablet/50 mL, Roche 11697498001), and 40 U/mL Protector Rnase Inhibitor (Roche 03335402001). The pellet was suspended in identical buffer, transferred to a 1.5 mL tube, and centrifuged at 20,817× *g* for 10 m at 4 °C. The supernatant was transferred to a fresh tube and frozen in liquid nitrogen. The ribosome preparation obtained from 90 g of wet yeast pellet was 122 mg/mL (27,120 pmol/mL). This procedure has been described in detail elsewhere [71]. As a quality control measure, 8000 pmol of the sample was analyzed by gradient centrifugation using 15–45% sucrose (Supplementary Material; Figure S6 and Table S2).

### 5.5. Synthesis of Defined ATA-Derived Analogs

Several ATA derivatives of defined length and molecular weight were synthesized in our laboratory. The quality and purity of samples was verified by high performance liquid chromatography (HPLC), nuclear magnetic resonance (H^1^-NMR) and liquid chromatography tandem mass spectrometry (LC-MS/MS). The synthetic route of ATA-derivative **11** is delineated in Scheme 3 and may be summarized as follows. Bis-formylation of **5** prepared according to Cushman et al. [39] (a) with hexamethylene tetramine/triethylamine followed by reduction with LiBH4 (b) led to **6,** which in the presence of sulfuric acid could be coupled with two equivalents of **7** (c) to produce tetramer **8**. Upon catalytic reduction with 10% PdO and H2 (d), tetramer **8** yielded **9**. The latter, when subjected to the same series of reactions used to convert **8** to **10** via **9**, yielded the desired dichlorohexamer **11**. This oligomer exhibited excellent activity when tested in reticulocytes and as an inhibitor of SARS-CoV-2 RdRp. The synthesis and purification of each ATA derivative is currently in progress and specific details will be reported elsewhere.

## Figures and Tables

**Table 1:** Thermodynamic Binding Parameters for ATA–HSA Interactions.

Compound	K_a_ × 10^5^ (M^−1^)	K_d_ (µM)	*n* ^a^	ΔG (kcal·mol^−1^)	ΔH (kcal·mol^−1^)	TΔS (kcal·mol^−1^)
ATA	5.0 ± 0.1	6.7 ± 0.1	4.5 ± 0.5	−7.6 ± 0.1	−6.4 ± 0.2	1.2 ± 0.1
Dichlorohexamer	9.9 ± 0.5	1.0 ± 0.1	1.0 ±0.0	−8.0 ± 0.2	−15.1± 0.2	−7.1 ± 0.2

^a^ The stoichiometry (*n*) is calculated based on the molecular weight for a monomeric unit of ATA (M_w_ 422.34) and the dichlorohexamer (M_w_ 956).

## Data Availability

Not applicable.

## Supplementary Materials

The following supporting information can be downloaded at: https://www.mdpi.com/article/10.3390/life12060872/s1, Figure S1: UV/Vis Absorbance Spectrum of the ATA Polymer Standard (2.0 mM) Revealing Major and Minor Peaks at 310 and 530 nm, respectively. Figure S2: Impact of has on ATA Emission Fluorescence; Figure S3: Fluorescence Intensity of ATA in the Presence of Increasing HSA Concentrations; Figure S4: ITC Profile of ATA Dilution into Dialysate; Figure S5: Full Size Immunoblotting Corresponding to Figure 8; Figure S6: Fractions of Yeast 80S Ribosomes Obtained via Sucrose Gradient Centrifugation. Table S1: Densitometry Results for Figure 8 and Supplementary Figure S6; Table S2: Optical Density of Yeast 80S Ribosome Fractions Obtained via Sucrose Gradient Centrifugation.

## Abbreviations

ATA: aurintricarboxylic acid (commercial heterogeneous preparation); CHX, cycloheximide; COVID, coronavirus disease; HTS, high throughput screening; PBS, phosphate buffered saline; RdRp, SARS-CoV-2 RNA dependent RNA polymerase (complex comprising SARS-CoV-2 nsp7, nsp8 and nsp12); SARS-CoV-2, severe acute respiratory syndrome coronavirus 2; SDS, sodium dodecyl sulphate.
