# Supplementary material for: Characterization of Aurintricarboxylic Acid (ATA) Interactions with Plasma Transporter Protein and SARS-CoV-2 Viral Targets: Correlation of Functional Activity and Binding Energetics
Source: Life (Basel). 2022 Jun 10;12(6):872. doi: 10.3390/life12060872 (PMC9227171; doi:10.3390/life12060872)
Supplement: Supplementary file 1 [file life-12-00872-s001.zip › life-1720422-supplementary.pdf]

Characterization of Aurintricarboxylic Acid (ATA) Interactions with Plasma Transporter Protein and SARS-CoV-2 Viral Targets: Correlation of Functional Activity and Binding Energetics

Conceição A. Minetti[1,#,*], David P. Remeta[1,#,*], Keiji Hashimoto[2], Radha Bonala[2], Rajesh Chennamshetti[2], Xingyu Yin[2], Miguel Garcia-Diaz[2], Arthur P. Grollman[2,3], Francis Johnson[2,4,*], and Viktoriya S. Sidorenko[2,*]

[1] Department of Chemistry and Chemical Biology; Rutgers - The State University of New Jersey; New Jersey, 08854; USA

[2] Department of Pharmacological Sciences, State University of New York at Stony Brook; Stony Brook; New York, 11794; USA

[3] Department of Medicine, State University of New York at Stony Brook; Stony Brook; New York, 11794; USA

[4] Department of Chemistry, State University of New York at Stony Brook; Stony Brook; New York, 11794; USA

SUPPORTING INFORMATION

SUPPLEMENTARY FIGURES

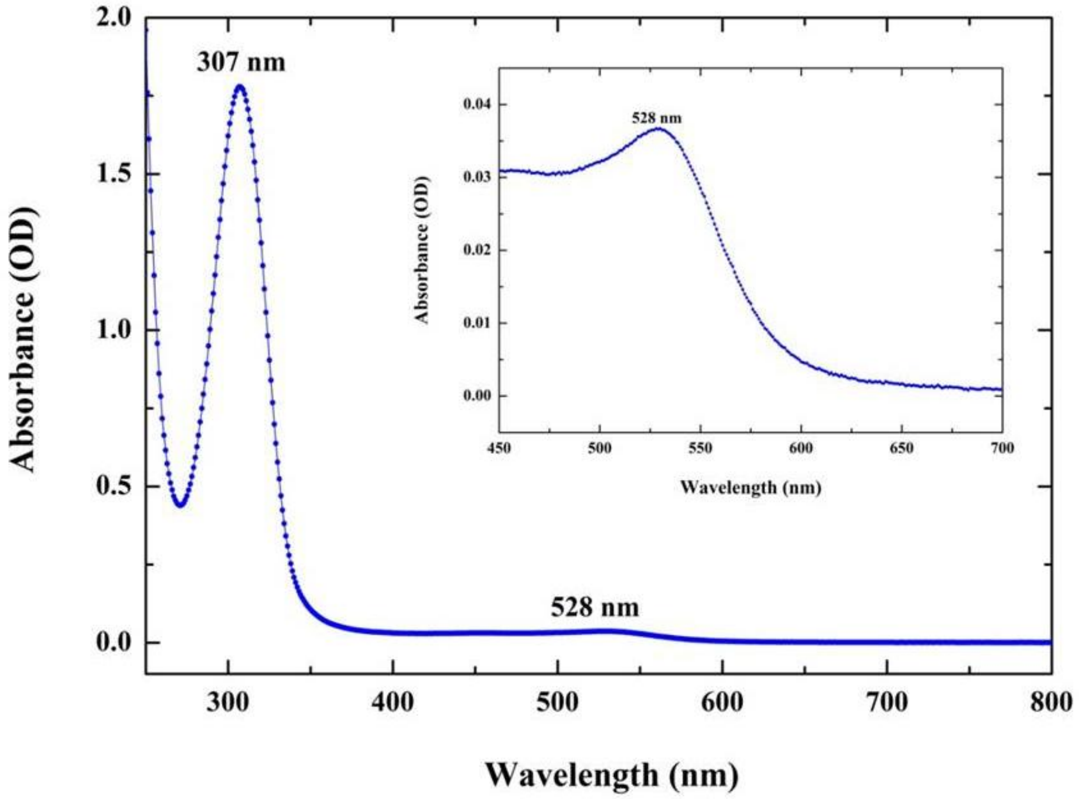

Figure S1. **UV/Vis Absorbance Spectrum of an ATA Polymer Standard (2.0 mM) Revealing Major and Minor Peaks at 310 and 530 nm, respectively.** The minor absorption band is expanded in the visible range (inset) to improve visualization.

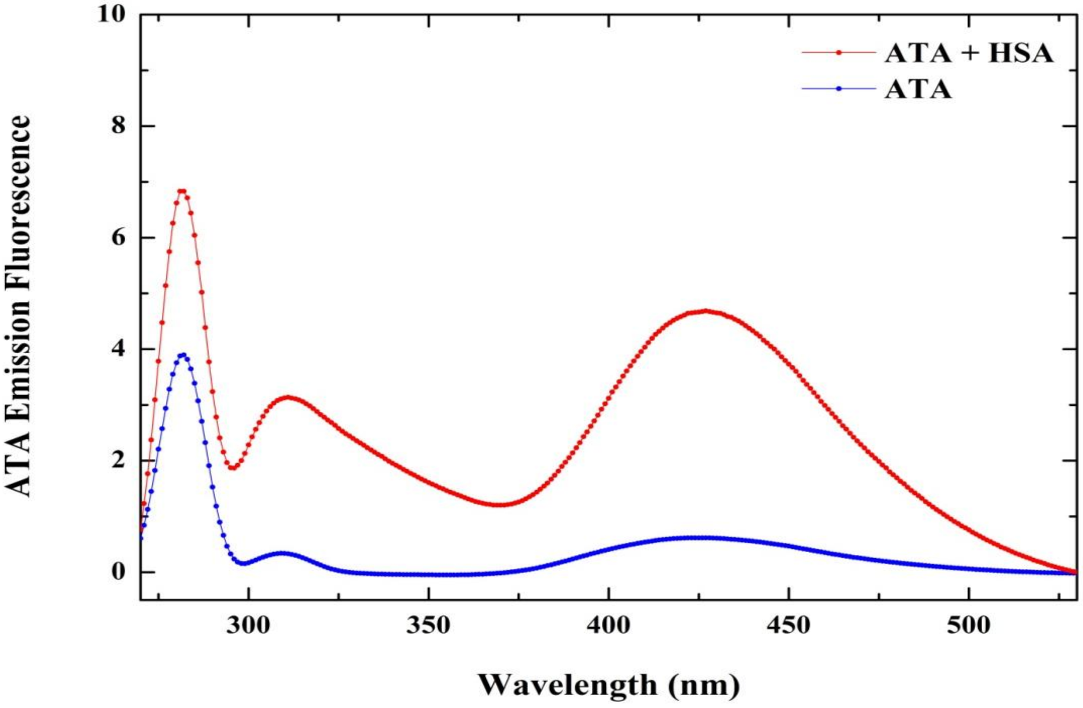

Figure S2. Impact of HSA on ATA Emission Fluorescence upon Excitation of Aromatic Residues at 280 nm. The enhanced ATA fluorescence intensity presumably arises as a consequence of energy transfer which can be exploited to assess ATA interactions at protein binding sites.

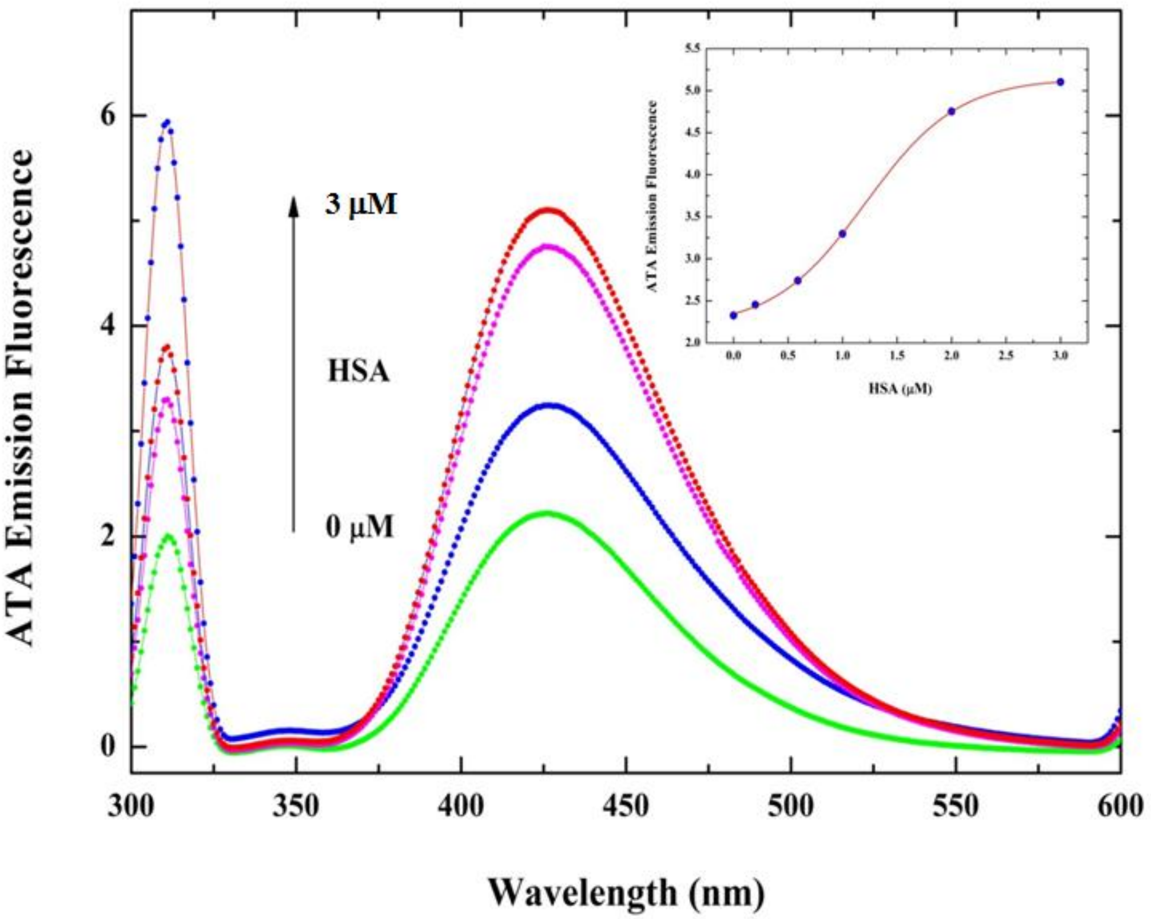

Figure S3. Fluorescence Intensity of ATA (λ_{Exc} 310 nm) in the Presence of Increasing HSA Concentrations Ranging from 0 to 3 μM. Inset: Fluorescence binding profile monitored at 425 nm as a function of HSA concentration.

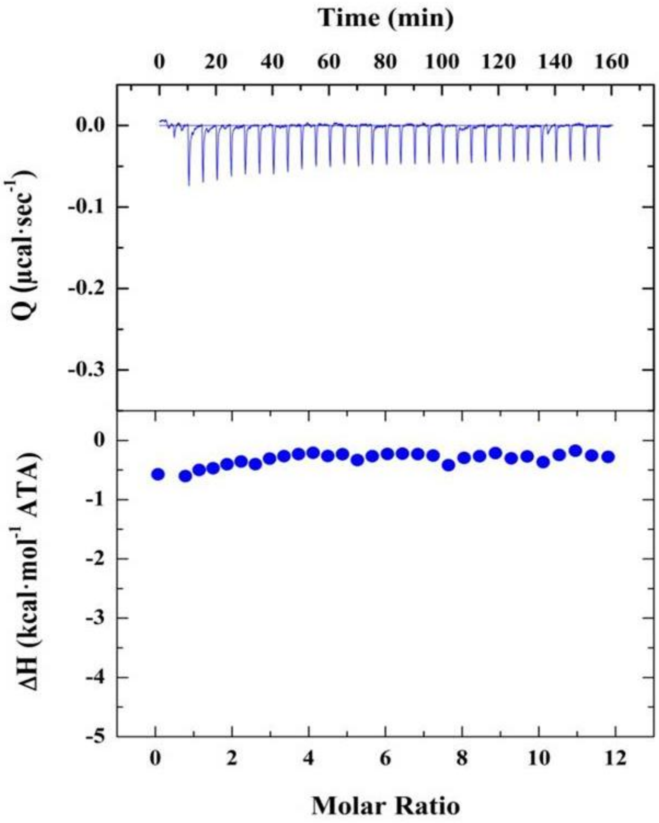

Figure S4. ITC Profiles Monitoring ATA Dilution into Dialysate. The binding isotherms are acquired at 25.0 ºC in 10 mM sodium phosphate (pH 7.4). Titration of ATA into dilaysate is reflected in the thermogram (top panel), integrated peak areas (blue circles) (bottom panel). The dilution of ATA into dialysate is characterized by negligible reaction heats reflecting the absence of aggregated species.

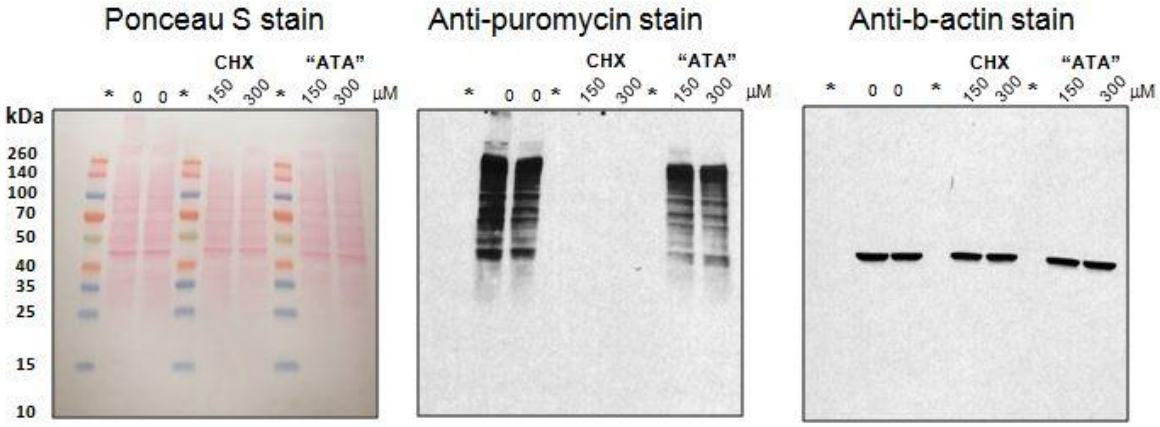

Figure S5. Full Size Immunoblotting Images Corresponding to Figure 8. The nitrocellulose membrane has been stained with Ponceau S to visualize total protein (left), anti-pyromycin antibodies (center), and anti-ß-actin antibodies (right).

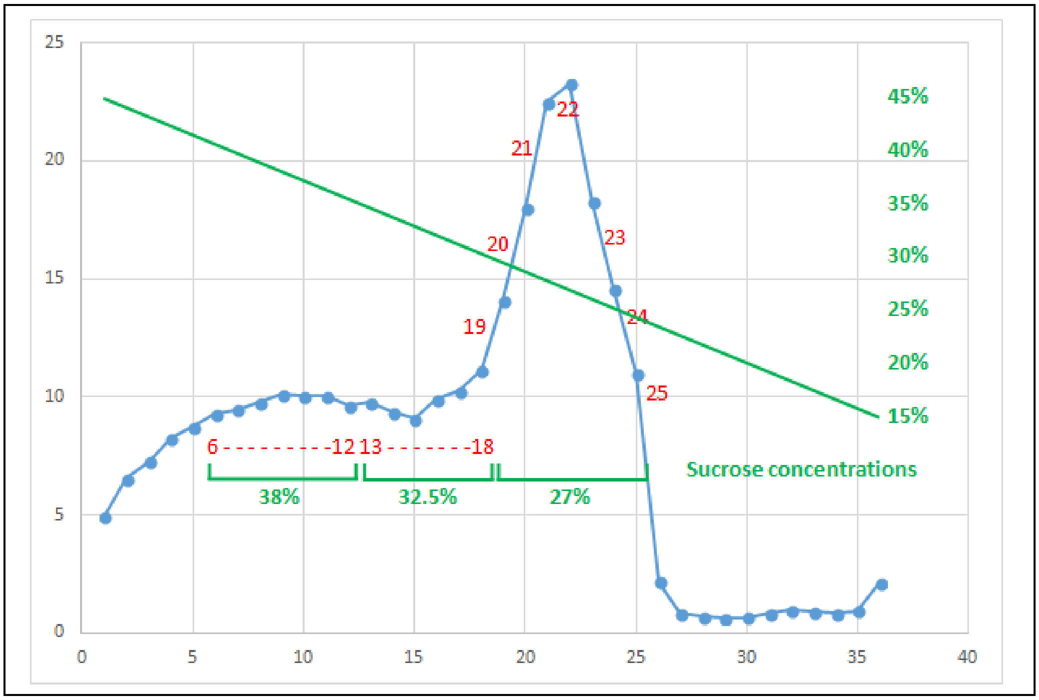

Figure S6. Fractions of Yeast 80S Ribosomes Obtained via Sucrose Gradient Centrifugation. Fractions 6-12 are polysomes while fractions 19-25 were combined and designated as 80S ribosomes. The latter were subsequently concentrated by sucrose cushion ultracentrifugation for thermodynamic studies.

Supplementary Tables

	b-actin densitometry								
Sample ID	Area	Mean	Min	Max	Max-Mean	Background adjusted			
Background	0.035	226.886	204	252	25.114				
Vero E6 no drugs_1	0.035	123.147	0	248	124.853	99.739			
Vero E6 no drugs_2	0.035	132.429	0	245	112.571	87.457			
Vero E6 CHX 150 uM	0.035	136.791	0	255	118.209	93.095			
Vero E6 CHX 300 uM	0.035	133.097	0	248	114.903	89.789			
Vero E6 ATA 150 uM	0.035	129.246	0	255	125.754	100.64			
Vero E6 ATA 300 uM	0.035	129.697	0	248	118.303	93.189			
	Anti-puromycin densitometry								
Sample ID	Area	Mean	Min	Max	Max-Mean	Background adjusted	Anti-puro/ b-actin	Reference	% activity
Background	0.376	226.557	180	255	28.443				
Vero E6 no drugs_1	0.376	126.97	6	247	120.03	91.587	0.918266676	0.910424	100.8615
Vero E6 no drugs_2	0.376	147.62	8	255	107.38	78.937	0.902580697		99.13853
Vero E6 CHX 150 uM	0.376	229.941	200	255	25.059	-3.384	-0.036349965		-3.99264
Vero E6 CHX 300 uM	0.376	229.785	202	255	25.215	-3.228	-0.035950952		-3.94882
Vero E6 ATA 150 uM	0.376	171.641	17	252	80.359	51.916	0.515858506		56.66137
Vero E6 ATA 300 uM	0.376	172.519	17	255	82.481	54.038	0.579875307		63.69291

Table S1. Densitometry results for **Figure 8** and Supplementary **Figure S6**.

Fraction	A260	Fraction	A260	Fraction	A260
1	4.988	13	9.770	25	11.027
2	6.525	14	9.344	26	2.191
3	7.273	15	9.072	27	0.843
4	8.254	16	9.922	28	0.716
5	8.728	17	10.258	29	0.666
6	9.315	18	11.159	30	0.680
7	9.473	19	14.110	31	0.860
8	9.787	20	18.004	32	0.999
9	10.137	21	22.519	33	0.934
10	10.083	22	23.284	34	0.857
11	10.046	23	18.272	35	0.950
12	9.663	24	14.594	36	2.150

Table S2. Optical Density of Yeast 80S Ribosome Fractions via Sucrose Gradient Centrifugation.
